# RNA triplet repeats: improved algorithms for structure prediction and interactions

**DOI:** 10.1186/s13015-025-00292-8

**Published:** 2025-12-10

**Authors:** Kimon Boehmer, Sarah J. Berkemer, Sebastian Will, Yann Ponty

**Affiliations:** 1 Laboratoire d’Informatique de l’Ecole Polytechnique (LIX CNRS UMR 7161), France, Institut Polytechnique de Paris, 1 Rue Honoré d’Estienne d’Orves, Palaiseau, 91120 France; 2 Earth-Life Science Institute, Institute of Science Tokyo, 2–12-1-I7E-318 Ookayama, Tokyo, 152–8550 Japan

**Keywords:** RNA folding, RNA interactions, Triplet repeats, Dynamic programming, NP-hardness

## Abstract

RNAs composed of Triplet Repeats (TR) have recently attracted much attention in the field of synthetic biology. We study the mimimum free energy (MFE) secondary structures of such RNAs and give improved algorithms to compute the MFE and the partition function. Furthermore, we study the interaction of multiple RNAs and design a new algorithm for computing MFE and partition function for RNA-RNA interactions, improving the previously known factorial running time to exponential. In the case of TR, we show computational hardness but still obtain a parameterized algorithm. Finally, we propose a polynomial-time algorithm for computing interactions from a base set of RNA strands and conduct experiments on the interaction of TR based on this algorithm. For instance, we study the probability that a base pair is formed between two strands with the same triplet pattern, allowing an assessment of a notion of orthogonality between TR.

## Introduction

RNAs composed of Triplet Repeats (TR) have attracted much attention, and harbour promises in the field of synthetic biology, due to their demonstrated capacity to self-assemble into droplets [1, 2]. Those can in turn be used to compartmentalize cellular processes, thereby creating a “clean room”, free of the natural cellular clutter, where synthetic circuits can be executed without interference. The exact process underlying this phenomena is still the object of ongoing investigations, but it is hypothesized that repetitive RNAs may induce Liquid-Liquid Phase separation mediated by unstable/transient structures. Repetitive RNAs are also found at the origin of severe Neurological Triplet Expansion Diseases (TED), including Friedreich attaxia [3] and Triplet Repeat Diseases (TRD) such as Huntington disease [4]. For multiple TEDs and TRDs, overly expanded RNAs have been observed to aggregate into RNA foci, leading to a sequestration of RNA binding proteins. Local secondary structures and interactions are impacted by the repeat, and generally believed to contribute to the pathogenicity and treatment efficiency. To study those phenomena *in silico*, and in particular the impact of the repeated motif and number of repeats on aggregates, one needs to predict the MFE structure of potentially large RNAs, and many-body interactions. Recently, coarse-grained simulations showed a disparity between odd or even numbers of triplet repeats [5] as well as extensions to quadruplet and non-redundant tandem repeats [6].

RNA folding by energy minimization is a classic algorithmic problem in Bioinformatics, historically solved in time $\Omega (n^3)$ using dynamic programming [7, 8]. Despite recent suggestions for heuristics [9], the best algorithm to date to solve energy minimization has runtime $\mathcal {O}(n^{2.8603})$ [10], and both its implementation and extension beyond a base-pair maximization setting represent considerable challenges. Prior works have also investigated conditional lower bounds, and found that the existence of a $\mathcal {O}(n^{2-\varepsilon })$ algorithm would refute the Strong Exponential Time Hypothesis (SETH) [10]. Meanwhile, an $\mathcal {O}(n^{\omega -\varepsilon })$ algorithm would disprove the *k*-clique conjecture, with $\omega <2.373$ being the matrix multiplication exponent [10, 11].

RNA-RNA interaction prediction represents an equally relevant, yet computationally substantially more involved algorithmic problem. For a fixed number of interacting strands, polynomial-time algorithms have been proposed. For example, by excluding so-called zig-zag joint conformations, Alkan et al. [12] proposed a polynomial-time algorithm for the interaction of two strands, while also showing **NP**-hardness for the case where we include these conformations. In the unbounded case, Dirks et al. [13] gave a factorial-time algorithm for computing the partition function (PF) over multiple strands. Additionally, it was shown that energy minimization in this setting is **APX**-hard (and by that **NP**-hard) [14], even for a very simple energy model. The problem is very much the object of ongoing investigations at different level of granularities, with striking recent results [15].

In this work, we show that the repeated nature of RNA can be exploited to obtain substantially improved algorithms for several problems. First, we show that the MFE of a triplet-repeat RNA can be predicted in linear time, both with respect to base pair maximization and Turner energy model, and is realized by either the open chain or a single helix. By a change of algebra, the DP scheme can be used to calculate the partition function. We then consider the interaction of multiple triplet repeats and propose improved algorithms for the general (non-triplet) case as well as algorithms specifically for the interaction of triplet repeats. For the latter case, we show **NP**-hardness in a reasonable energy model. We then propose a polynomial-time algorithm for the setting where we are given a “soup” of strands instead of a fixed set, and, using this algorithm, conduct experiments on the probability that a base pair is folding, interacting with another identical sequence or interacting with a different sequence.

## Definitions and problem statement

### Definitions

**RNA sequence and folding.** An *RNA sequence* (or just *sequence*) is a word $s \in \{\texttt {A},\texttt {C},\texttt {G},\texttt {U}\}^+$. The length of *s* is denoted by |*s*| and the *i*-th position of *s* by $s_i$. A position on a sequence is also called a *base*. We associate to each base $s_i$ its letter by $l(s_i)$. We define $P:=\{\{\texttt {C},\texttt {G}\},\{\texttt {A},\texttt {U}\},\{\texttt {G},\texttt {U}\}\}$ as the set of *admissible base pairs*. A *(pseudoknot-free) secondary structure* *S* is a set of unordered pairs of bases, hereunder called *base pairs*, such that:

- each base pair is a Watson-Crick or Wobble pair, i.e. for all $\{s_i,s_j\} \in S$, $\{l(s_i),l(s_j)\} \in P$;
- each base is involved in at most one base pair, i.e. for all bases $s_i$, $|\{p \in S \mid s_i \in p\}| \le 1$;
- *S* is *pseudoknot-free*, i.e. there are no $\{s_i,s_j\},\{s_k,s_\ell \} \in S$ with $i<k<j<\ell$;
- each base pair encloses at least $\theta$ bases, i.e. if $\{s_i,s_j\}\in S$, then $j-i>\theta$. The *minimal base pair span* is usually denoted by $\theta$, and we use $\theta :=3$ unless explicitly specified.

We denote by $\Omega (s)$, or in short $\Omega$ whenever clear from the context, the set of all pseudoknot-free secondary structures over sequence *s*.

We associate each secondary structure $S \in \Omega$ to a *free energy*, according to an *energy model* $E: \{\texttt {A},\texttt {C},\texttt {G},\texttt {U}\}^+ \times \Omega \rightarrow \mathbb {R}$. For example, in the *base pair model* $E_\text {bp}$, we simply count the number of base pairs in *S*, hence set $E_\text {bp}(s,S) = -|S|$. More advanced energy models reason about the free energy introduced by motifs occurring in the secondary structure, such as the loops considered by the Turner nearest-neighbor model [16].

**Interactions.** A strand is an RNA sequence which is identified as a unique object in a set. In other words, in a set of strands *R*, we can have two strands $s \ne r$ that consist of the same sequences, that is $l(s_i)=l(r_i)$ for all $i \in \{1,...,|s|=|r|\}$, but still are different objects. To describe the interaction of multiple strands, we are given a set *R* of strands, where $m:=|R|$.

A *circular permutation* $\pi :R \rightarrow \{0,...,m-1\}$ of a strand set *R* is a permutation of all elements in *R* except for one fixed strand $s^*$, which is fixed to position 0. Then, the bases are naturally ordered by $s_i<_\pi r_j \equiv s<r \vee (s=r \wedge i<j)$. We define $O_\pi$ as the set of all tuples of bases $(s^1_{i_1},...,s^k_{i_k})$ such that there is a *j* with $s^j_{i_j}<_\pi s^{j+1}_{i_{j+1}}<_\pi ...<_\pi s^k_{i_k}<_\pi s^1_{i_1}<_\pi ...<_\pi s^{j-1}_{i_{j-1}}$.

A *secondary structure* *S* of a strand set *R* is a set of base pairs $\{s_i,r_j\}$ from strands in $s,r \in R$ such that $\{l(s_i),l(r_j)\} \in P$, each base appears in at most one base pair and each intra-strand base pair encloses at least $\theta$ bases, i.e. $\{s_i,s_j\} \in S \rightarrow j-i>\theta$.

**Fig. 1:**
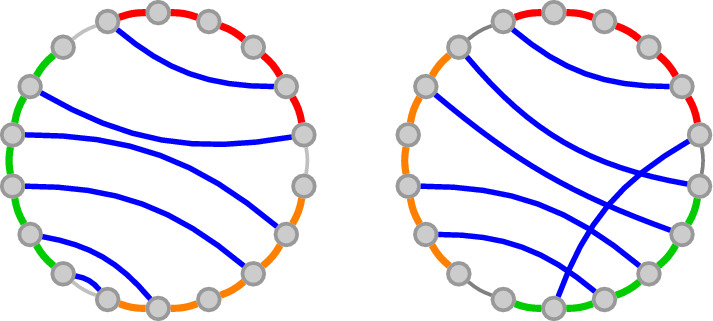
The same secondary structure on a strand set with three strands drawn in two different circular permutations. The strands are depicted by the green, red and orange lines while blue lines indicate base pairs. Gray lines connect subsequent strands and depend on the strand permutation

The *polymer graph* of a secondary structure *S* and a circular permutation $\pi$ on *R* is a graph $G=(V,E)$ with $V:=\{s_i \mid s \in R, 1 \le i \le |s|\}$ and $E:=S \cup \{\{s_i,s_{i+1}\} \mid s \in R, 1 \le i < |s|\} \cup C:=\{\{s_{|s|},r_1\} \mid (\pi (s)+1) \bmod |R| = \pi (r)\}$. The edges $E-S$ are drawn in a cycle (naturally induced by the circular permutation), while the edges in *S* are drawn as straight lines between the bases. Examples for the polymer graphs of a single secondary structure under two different circular permutations can be found in fig. 1.

Two strands *s*, *r* are *connected* if there is a path from $s_1$ to $r_1$ that does not use edges from *C*. A secondary structure is connected if all of its strands are connected. Note that connectedness is independent of the circular permutation $\pi$.

A secondary structure *S* of a strand set *R* is called *pseudoknot-free* if there is a circular permutation $\pi$ such that there are no crossing lines in the polymer graph, or formally, there are no two base pairs $\{s_i,t_k\},\{u_\ell ,r_j\} \in S$ with $(s_i,u_\ell ,t_k,r_j)\in O_\pi$. The set of all pseudoknot-free secondary structures over a strand set *R* is denoted by $\Omega (R)$.

As for the folding, we associate to each $S \in \Omega (R)$ a free energy $E:2^{\{\texttt {A},\texttt {C},\texttt {G},\texttt {U}\}^*} \times \Omega \rightarrow \mathbb {R}$. In the base pair model, apart from the number of base pairs *p* of base pairs, we also add a strand association penalty $K_\text {assoc}$ for each of the $(m-\ell )$ strand associations, where $\ell$ is the number of connected components (also called *complexes*) of *S*. Thus, the free energy of $S \in \Omega$ in this model is defined as $E(R,S)=-p+(m-\ell )K_\text {assoc}.$

### Computational problems

For a single strand, two of the most classical problems in RNA bioinformatics are: 
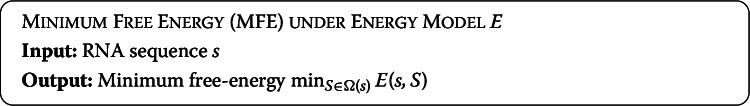

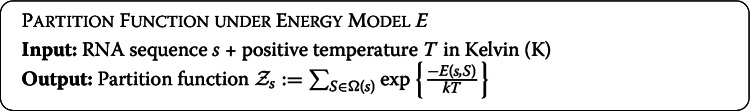
 where $k=1.987 \cdot 10^{-3} \text {kcal}.\text {mol}^{-1}.\text {K}^{-1}$ is the Boltzmann constant.

In the multi-strand setting, we focus on energy minimization. In Dirks et al. [13], the authors adopt a thermodynamic perspective on the free energy of a secondary structure over multiple strands, such that potential rotational symmetries require an adjustment of the computed value. For the MFE, we focus on a more algorithmic perspective, where all rotationally symmetric structures are elements of a search space, and a simple base pair energy model. In our main algorithmic problem of interest, we are given a set of strands and are looking for the minimum free energy of the secondary structure over these strands: 
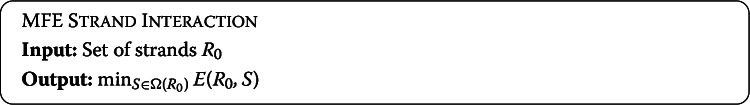
 In a more applied setting in the field of synthetic biology, we consider a set of RNA strands composed out of triplet repeats to be present such that a sufficient subset thereof will self-assemble into droplets. Hence, we assume having a ’soup’ of RNA strands in question as a basis for the formation of RNA droplets that will serve as point of departure.

Therefore, we consider here a slightly different setting, where the number of occurrences of each triplet/strand is unconstrained beyond the total number *m* of interacting strands. This allows to study situations where the strands concentrations are in excess, so that sequences can be locally seen as infinitely available often within a set (or “soup”) *R* of strands. We then look for the best structure over *m* strands that all appear in *R*. Each sequence in the soup can appear zero or multiple times in a secondary structure. More formally: 
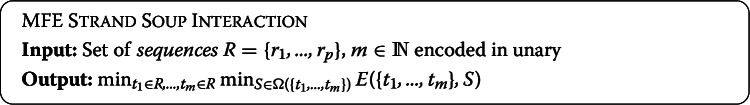


### Triplet repeats RNAs and their properties

**Triplet repeat RNAs (TR).** Of special interest to us are RNA sequences that are composed of *triplet repeats* (TR), that is, they have the form $(X \cdot Y \cdot Z)^k$ for $X,Y,Z \in \{\texttt {A},\texttt {C},\texttt {G},\texttt {U}\}$ and $k \in \mathbb {N}^+$. We will describe how we can improve the general algorithms for the above computational problems in the case of TR.

**Fig. 2:**
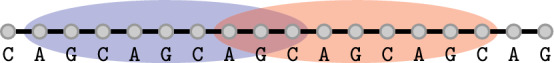
The periodicity of triplet repeats RNAs (TR) induces multiple symmetries, leading to drastic simplifications of certain algorithmic problems. Here, the blue and red regions of the TR sequence are identical

An algorithmically consequential property of any region $[s_i,s_j]$ in a TR sequence is the following.

#### Observation 1

For a triplet repeat sequence *s* and $1 \le i \le j \le |s|$, one has

$$\begin{aligned} {[}s_i,s_j]=[s_{i \bmod 3}, s_{j - (i - i \bmod 3)}]. \end{aligned}$$

In other words, we can shift any region three positions to the left or right, and in particular we can shift it to the beginning of the sequence, as visualized in Fig. 2. That way, the index that usually denotes the beginning of the considered sequence in a dynamic programming (DP) algorithm can be restricted to values 1, 2 and 3. Hence, the length of the value range is constant and not linear anymore, which gives an easy linear improvement of running time and storage for MFE as well as PF computation.

We also note that TR sequences can be encoded exponentially more compact than general sequences. Each TR sequence is uniquely identified by its pattern $XYZ \in \{\texttt {A},\texttt {C},\texttt {G},\texttt {U}\}^3$ and its number of repeats *k*. In other words, $6+\lceil \log _2 k \rceil$ bits are enough to encode a TR sequence with *k* repeats. We will refer to this encoding as the *compact encoding*, while the *explicit encoding* consists of the complete sequence $s \in \{\texttt {A},\texttt {C},\texttt {G},\texttt {U}\}^{3k}$. The latter can also be seen, equivalently in terms of asymptotic complexity, as a compact encoding where *k* is encoded in unary.

Looking into more structural properties of triplet repeats, we can observe that, since each base repeats after two other bases, there cannot be a base pair that encloses exactly 2 bases. Thus, requiring two ($\theta =2$) or three ($\theta =3$) enclosed bases between any base pair is equivalent:

#### Observation 2

A secondary structure *S* for $(XYZ)^k$ fulfills minimum base pair span $\theta$ with $\theta \equiv _3 2$ if and only if it fulfills minimum base pair span $\theta +1$.

Finally, if we consider the graph $G=(\{\texttt {A},\texttt {C},\texttt {G},\texttt {U}\},P)$, where *P* is the set of allowed base pairs, we can see that it does not contain any triangles. From this we can observe:

#### Observation 3

For any triplet sequence $(XYZ)^k$, there is a letter $V \in \{X,Y,Z\}$, that we call the *covering letter*, that is contained in all base pairs, i.e. $V \in p$ for all $p \in S$ and $S \in \Omega$.

## Single-stranded triplet repeats

Our goal is to specify the exact MFE, and the corresponding secondary structure, when given a triplet pattern *XYZ* and length *k* of our TR sequence *s*, as well as the minimum base pair span $\theta$. This will give us a very efficient way of computing the MFE in this simple setting.

### Linear time solution for base pair maximization

We first consider the properties of the MFE structure for TR RNAs in a *base pair maximization model*, where the free energy $E_\text {bp}$ of a secondary structure $S \in \Omega$ is such that $E_\text {bp}(s,S) = -|S|$.

We can first prove an upper bound on the number of base pairs in a TR sequence:

#### Lemma 1

Consider a TR sequence $s:=(XYZ)^k$ and a minimum number of enclosed bases $\theta \ge 0$, such that $\lfloor \frac{\theta +1}{3} \rfloor \le k$. We have $E_\text {bp}(s,S) \le k-\lfloor \frac{\theta +1}{3} \rfloor$ for any $S \in \Omega (s)$.

#### Proof

Without loss of generality, let *Z* be the covering letter of *s*. Any non-empty secondary structure has an innermost base pair which must respect the minimum base pair span $\theta$. For $\theta =2$, which is equivalent to $\theta =3$ by observation 2, as well as for $\theta =4$, at least one *Z* base must remain unpaired, and increasing $\theta$ by 3 will result into one new unpairable *Z* base. Thus we know that at least $\lfloor \frac{\theta +1}{3} \rfloor$ *Z* bases will remain unpaired and at most $k-\lfloor \frac{\theta +1}{3} \rfloor$ *Z*-bases are pairable. Since every base pair must involve a *Z* base, we can conclude. $\square$

We now show that this upper bound is almost always tight. To this end, first notice that for all triplet patterns *XYZ* such that $\{\{X,Y\},\{X,Z\},\{Y,Z\}\} \cap P = \varnothing ,$ no base pair can be built and thus the maximum value is trivially 0. We call TR sequences of such patterns *non-folding*, and all other TR sequences *folding*.

#### Lemma 2

For $\theta \in \{0,1\}$ and $k>1$, we always have $E(s,S)=k$ for any secondary structure *S* over a folding sequence $s=(XYZ)^k$.

#### Proof

If $\{X,Z\} \in P$, connect *X* and *Z* in each triplet. Else, connect the outermost pair (say without loss of generality $\{X,Y\}$). We obtain the inner sequence $(YZX)^{k-1}$ (with $k-1 > 0$) and we can proceed as above since $\{Y,X\} \in P$. $\square$

For the more natural case $\theta > 1$, the upper bound from lemma 1 is not always tight. The next lemma exactly specifies the MFE and its structure:

#### Lemma 3

Let $\theta >1$. The MFE structure of a folding sequence $(XYZ)^k$ has value

- $$k-1 - \frac{\theta -1}{3}$$, if $$(\{X,Z\}\not \in P \wedge (\theta + 3k)\equiv _6 4) \vee (\{X,Y\},\{Y,Z\}\not \in P \wedge (\theta +3k) \equiv _6 1)$$
- $$k - \lfloor \frac{\theta +1}{3} \rfloor $$, otherwise;

where $\equiv _6$ is used to denote the 6 different equivalence classes we obtain from the possibilities to form base pairs between two given triplets. Furthermore, a MFE structure is obtained by a single helix of base pairs of one letter pair *p*. If both $\{X,Z\} \in P$ and one of $\{X,Y\}$ and $\{Y,Z\} \in P$, we set $p:=\{X,Z\}$ if $(\theta +3k) \equiv _6 4$ and $p:=\{X,Y\}$ (or $p:=\{Y,Z\}$) if $(\theta +3k) \equiv _6 1$; otherwise, we set *p* to the letters of an arbitrary pairable base pair.

To improve readability of the manuscript, the proof of Lemma 3 has been moved to the supplemental material.

Setting $\theta =3$, we get the following corollary:

#### Corollary 1

In the base pair maximization model, if $\theta =3$, the MFE structure of any TR sequence $(XYZ)^k$ has $k-1$ base pairs.

**Fig. 3:**
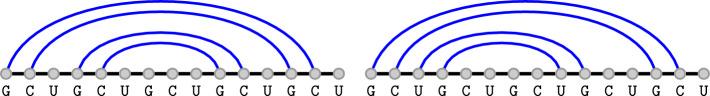
Two distinct optimal secondary structures for ${\texttt {GCU}}_5$, for $\theta =3$

Determining the MFE is thus a simple calculation taking logarithmic time in the (explicit) size of the triplet repeat sequence. From this we can derive:

#### Theorem 1

MFE prediction for compactly encoded TR in the base pair maximization model can be solved in linear time.

The structure itself can then be computed by Algorithm 1:

**Algorithm 1:**
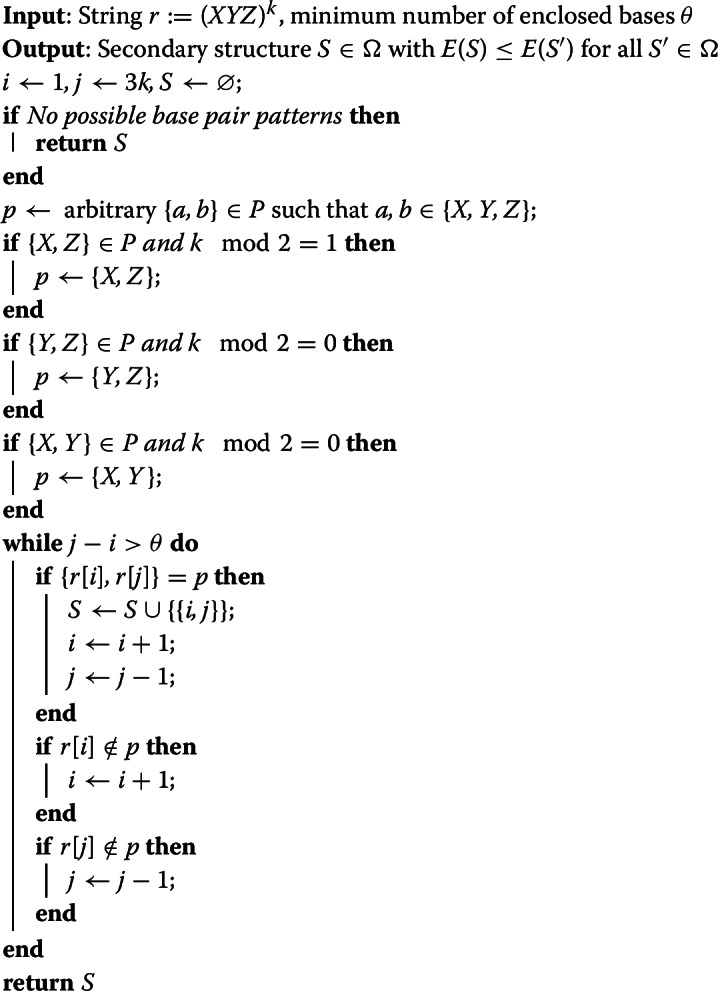
Computing the MFE of Triplet Repeat RNAs

The algorithm clearly runs in linear time, when the input is an explicit (unary) encoding of the triplet repeats. Its correctness directly follows from lemma 3.

#### Remark 1

The optimal secondary structure does not need to be unique. In particular, for a simple energy model, the number of optimal secondary structures for triplet repeats can even be exponential. For example, consider the sequence $({\texttt {GCU}})^k$ as illustrated in fig. 3. When constructing the base pairs from outside to inside, in every step, we can choose between adding the base pairs ${\texttt {G-U}},{\texttt {U-G}}$, or the base pairs ${\texttt {G-C}}, {\texttt {C-G}}$. This decision can be repeated $\lfloor \frac{k}{2} \rfloor -1$ times (assuming $\theta =3$), giving $\Omega (2^{{k}/{2}})$ different optimal secondary structures.

### Minimum free-energy in the Turner model

For the Turner model, we will argue that the optimal structures obtained for BP maximization remains optimal for the Turner nearest neighbor model under reasonable assumptions, satisfied by current versions of the model [16]. We first show a helpful lemma:

#### Lemma 4

Assume that a TR region of *s* where the covering letter appears *k* times has *B* branches. Then the number of base pairs is at most $k-B$.

#### Proof

Let *V* be the covering letter of *s*. By observation 3, for each base pair {$s_i,s_j\}$, either $l(s_i)=V$ or $l(s_j)=V$. Furthermore, each of the *B* branches contains one unpairable *V*-base (since $\theta =3$). Thus, there are only $k-B$ pairable *V*-bases and we conclude. $\square$

We show the absence of multiloops, i.e. structural motifs consisting of $B \ge 2$ branches, in the Turner MFE, with some simplifications. Their free energy contribution is composed of an initiation penalty $\alpha$, a value $\beta$ for each branch, and an asymmetry penalty $\gamma$. The overall contribution of a multiloop *S* is given by $E(s,S)=\alpha +\beta B+\gamma C + E_\text {in}$, where $E_\text {in}$ is the MFE of the interior secondary structure of the branches. We will assume $N:= \min _{V,W \in \{X,Y,Z\}: \{V,W\} \in P} E_{V,W}$ to be the best contribution of a single base pair appearing in a stacking in our triplet pattern, and we will not consider dangling ends etc.

#### Lemma 5

Any Turner-MFE secondary structure $S^*$ over a TR sequence does not contain any multiloop, assuming $\beta \ge N,\alpha > -\beta , \gamma \ge 0$.

#### Proof

Let *S* be a multiloop structure on region *s* with *k* appearances of the covering letter and let $S^*$ be a stacking on the same region. Their free energy values are related as follows:

1 $$\begin{aligned} E(s,S)&\ge \alpha + \beta B + \gamma C + (k - B)N \end{aligned}$$

2 $$\begin{aligned}&> -\beta + \beta B + (k-B) N \end{aligned}$$

3 $$\begin{aligned}&= (k-1)N + (\beta -N) (B-1) \end{aligned}$$

4 $$\begin{aligned}&\ge (k-1)N \end{aligned}$$

5 $$\begin{aligned}&\ge E(s,S^*) \end{aligned}$$

where (1) comes from our above observation and lemma 4, (2) from $\alpha >-\beta$ and $\gamma \ge 0$, (4) from $\beta \ge N$ and $B \ge 2$ (by definition of a multiloop). For inequality (5), first notice that $S^*$ contains $k-1$ base pairs by corollary 1. As noticed in remark 1, we can choose which base pair is used in $S^*$ without affecting the optimality. In particular, we can always choose the base pair consisting of the letters *V*, *W* that optimize their contribution, such that $E_{V,W}=N$. We get $E(s,S^*) \le (k-1)N$. $\square$

#### Remark 2

The above assumptions are satisfied by the Turner 2004 energy model ($\alpha =9.25$, $\beta =-0.63$, $\gamma =0.91$ and $N \le -0.93$), as seen in the NNDB [16].

lemma 4 also excludes secondary structures with multiple exterior faces. Thus, by the above two lemmata, we can conclude that the MFE in the Turner model is also of the canonical form described in the BP maximization setting.

### Linear-time computation of the partition function

In the context of computing the partition function, one can write a weighted context-free grammar which, for any given pattern *XYZ*, simultaneously generates all TR sequences along with their associated set of secondary structures $\Omega$.

As an example, a context-free grammar associated with the pattern CAG would be:

$$\begin{aligned} S_C^G&\rightarrow ~~~ (~\cdot _A~S_G^C~ \cdot _A ~)~&\mid&~ (~\cdot _A~ S_G^C~ \cdot _A ~) ~S_C^G ~&\mid&~\cdot _C ~\cdot _A ~S_G^G~&\mid&~\cdot _C~\cdot _A~\cdot _G\\ S_G^C&\rightarrow ~~~ (~S_C^G~)~&\mid&~(~S_C^G~)~\cdot _A~ S_G^C~&\mid&~ \cdot _G~ S_C^C ~&\mid&~ \cdot _G~ \cdot _C\\ S_G^G&\rightarrow ~~~ (~S_C^G~)~ \cdot _A~ \cdot _G~&\mid&~(~S_C^G~)~ \cdot _A~ S_G^G ~&\mid&~ \cdot _G~ S_C^G  &   \\ S_C^C&\rightarrow ~~~ (~ \cdot _A~ S_G^C~ \cdot _A~ )~ \cdot _A~&\mid&~ (~ \cdot _A~ S_G^C~ \cdot _A ~)~ S_C^C~&\mid&~ \cdot _C~ \cdot _A ~S_G^C  &   \end{aligned}$$

Here, the terminal $S_C^G$ generates all secondary structures for the RNA sequence $(CAG)^k$ for all $k>0$; $S_G^C$ the structures of $(GCA)^k GC$ for $k \ge 0$; $S_G^G$ the structure of $G(CAG)^k$ for $k>0$; and $S_C^C$ corresponds to the pattern $(CAG)^kC$ for some $k>0$.

Following standard methodologies in enumerative/analytic combinatorics [17], such a grammar can be generically translated into a system of functional equations involving weighted generated functions for each non-terminal:

$$\begin{aligned} S_C^G(z)&= \beta \,z^4\,S_G^C(z)+ \beta \,z^4\,S_G^C(z)\,S_C^G(z) + z^2\,S_G^G(z)+z^3\\ S_G^C(z)&= \beta \,z^2\,S_C^G(z) + \beta \,z^3\,S_C^G(z)\,S_G^C(z)+ z\, S_C^C(z) + z^2\\ S_G^G(z)&=\beta \,z^4\,S_C^G(z) + \beta \,z^3\,S_C^G(z)\,S_G^G(z) + z\, S_C^G(z) \\ S_C^C(z)&= \beta \,z^3\,S_G^C(z) + \beta \,z^2\,S_G^C(z)\,S_C^C(z) + z^2\,S_G^C(z) \end{aligned}$$

where $\beta :=e^{1/kT}$ is the Boltzmann weight associated to base pairs and, in particular:The partition function of $\mathcal {Z}_{(CAG)^k}$ can then be obtained as $[z^{3k}]\, S_C^G(z)$, the coefficient of degree 3*k* in $S_C^G(z)$. Since the system of functional equations is algebraic, the coefficients of each generating function obey a linear recurrence with polynomial coefficients [18], which can be efficiently [19] and effectively computed [20]. We obtain an equation of the form:

$$\begin{aligned} \mathcal {Z}_{(CAG)^k} = P_1(k)\, \mathcal {Z}_{(CAG)^{k-1}} + P_2(k)\, \mathcal {Z}_{(CAG)^{k-2}} + \cdots + P_d(k)\, \mathcal {Z}_{(CAG)^{k-d}}\end{aligned}$$

where each $P_i$ is a polynomial in *k*, and *d* is a constant. $\mathcal {Z}_{(CAG)^k}$ can then be computed using a linear number of arithmetic operations. This property holds for other triplets and thus:

#### Theorem 2

The partition function of a TR can be computed in $\Theta (k)$ arithmetic operations.

## Interaction of triplet repeats

We now consider a set $R_0$ of triplet repeat strands. Our goal is to find the minimum free energy secondary structure for $R_0$. We defined the computational problem MFE Strand Interaction in section 2.2. In the base pair maximization model, this gives exactly the same definition as in [14], where the authors show that the problem is **APX**-hard (and by that **NP**-hard) for the general (non-triplet) case. On the other hand, Dirks et al [13] gave a factorial-time algorithm for computing the partition function over multiple strands. In this section, we improve both results: we show that the problem is ${{\textbf {NP}}}$-hard in a reasonable energy model even if restricted to triplet repeats of one pattern; we give an exponential-time, instead of factorial, algorithm for the problem.

However, our exponential-time algorithm is designed for solving the MFE from an algorithmic perspective, as discussed in section 2.2, and does not account for the rotational symmetries to the free energy described by Dirks et al. [13]. Consequently, the DP scheme will not necessarily compute the MFE value in this model, although on a practical level it is likely that the symmetry-corrected structure can be found by investigating a small number of suboptimal structures. Moreover, for the partition function, we can account for the algorithmic overcounting and additionally, if desired, for penalties associated with rotational symmetries.

### General RNA–RNA interactions

The main difficulty of the problem lies in the fact that we need to consider all possible circular permutations of strands. Instead of trying all of these circular permutations one by one and applying a classical single-stranded folding algorithm, we build up the values for all possible circular permutations while exploring all possible joint secondary structures. More specifically, we will consider structures consisting of a leftmost strand and its position, a rightmost strand and its position, as well as a set of strands which have to appear in between the leftmost and rightmost strand (without specifying the ordering of these strands).

**Fig. 4:**
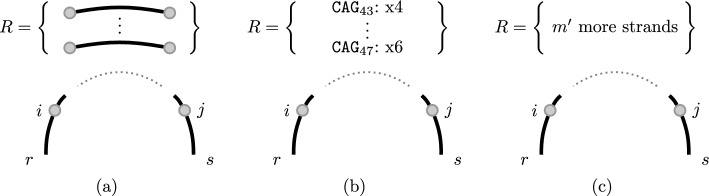
Visualization of the structures used to compute the MFE in the **a** general setting, **b** TR setting and **c** strand soup setting. The set of strands is represented explicitly in **a**. In **b**, we store for each appearing sequence (e.g. $\texttt {CAG}_{43}$) the number of appearances (e.g. x4). In **c**, we only store the number of strands between *r* and *s*

We can formulate DP recurrences as follows: Let $E_{s_i,r_j}$ be the minimum free energy induced by the base pair between the *i*-th base of strand *s* and the *j*-th base of strand *r*. In our DP equations, $R \subseteq R_0$ denotes the subset of still available strands, $s \in R$ the leftmost strand, $r \in R$ the rightmost strand, $1 \le i \le |s|$ the current position in *s*, $1 \le j \le |r|$ the current position in *r*, and $c \in \{0,1,2\}$ indicates whether *s* and *r* will be connected by a base pair (0: no base pair allowed, 1: at least one base pair required, 2: a base pair is not required; if the left and right strand are equal, then $c=2$). The structures with which our algorithm works are visualized in Fig. 4a. The main recurrences are as follows:

$$\begin{aligned} M_{R,s_i,r_j,c} = \min {\left\{ \begin{array}{ll} {\left\{ \begin{array}{ll} M_{R,s_{i+1},r_j,c} & \text {if }i+1\le |s| \\ \min _{t \in R, c' \in \{0,1\}} M_{R - \{s\},t_1,r_j,c'}- \mathbb {1}_{c'=0}K_\text {assoc}& \text {if }i+1>|s|\text { and }c\ne 1\\ +\infty & \text {else} \end{array}\right. }\\ {\left\{ \begin{array}{ll} E_{s_i,r_j}+\bar{M}_{R,s_i,r_j,2} & \text {if }c\ne 0\\ +\infty & \text {if }c=0 \end{array}\right. }\\ \min _{R',t,k} E_{s_i,t_k}+\bar{M}_{R',s_i,t_k,2}+\bar{M}_{(R-R') \cup \{s\},t_k,r_{j+1},c} \end{array}\right. } \end{aligned}$$

where

$$\begin{aligned} \bar{M}_{R,s_i,r_j,c}={\left\{ \begin{array}{ll} M_{R,s_{i+1},r_{j-1},c} & \text {if }i+1 \le |s|\text { and }j-1 \ge 1\\ \min _{t \in R-\{s,r\}, c' \in \{0,1\}} M_{R - \{s,r\},t_1,r_{j-1},c'}- \mathbb {1}_{c'=0}K_\text {assoc} & \text {if }i+1 > |s|\text { and }j-1 \ge 1 \\ \min _{u \in R-\{s,r\}, c' \in \{0,1\}} M_{R - \{s,r\},s_{i+1},u_{|u|},c'}- \mathbb {1}_{c'=0}K_\text {assoc} & \text {if }i+1 \le |s|\text { and }j-1 < 1 \\ \min _{t,u \in R-\{s,r\}, c' \in \{0,1\}} M_{R - \{s,r\},t_1,u_{|u|},c'} - \mathbb {1}_{c'=0}K_\text {assoc} & \text {else}\\ \end{array}\right. } \end{aligned}$$

and $-K_\text {assoc}$ is a reward for an additional complex. We give this reward each time we “choose” a new strand from *R* and decide that it should not be connected to the other extremity of the interval ($c'=0$). The $\bar{M}_{R,s_i,r_j,c}$ equation gives the MFE for the region $]s_i,r_j[$ (i.e. $[s_{i+1},r_{j-1}]$ if $i+1 \le |s|$ and $j-1 \ge 1$, and introducing new strands in the other cases).

Choosing an arbitrary strand *s*, the minimum free energy can be finally computed by

$$\begin{aligned} E^*(R)=(m-1) \cdot K_\text {assoc} + \min _{r \in R - \{s\},c\in \{0,1\}} M_{R,s_1,r_{|r|},c} \end{aligned}$$

and the optimal secondary structure can be obtained through backtracking.

For the initialization, we can set $M_{\{s\},s_i,s_j}=0$ for valid indices $j-i \le \theta$ for any $s \in R$. We now prove that $M_{R,s_i,r_j}$ is computed correctly. By slight abuse of notation, we write $s_i \in S$ for $s_i \in \bigcup _{P \in S} P$.

#### Definition 1

An *interval* for this DP is denoted by $[R,s_i,r_j,c]$ where $s,r \in R$, $1 \le i \le |s|$, $1 \le j \le |r|$ and $c \in \{0,1,2\}$. An interval $[R',t_k,u_\ell ,c']$ is *included* in interval $[R,s_i,r_j,c]$, written $[R',t_k,u_\ell ,c'] \preccurlyeq [R,s_i,r_j,c]$, if one of the following holds:

- $$R' \subset R$$ and $$|R'| < |R| -1$$
- $$R' \subset R$$, $$|R'| = |R| -1$$ and $$s=t \vee r=u$$
- $$R'=R$$, $$s=t$$, $$r=u$$, $$i \le k$$ and $$\ell \le j$$.

If we replace *both* inequalities by strict inequalities in the last point, the interval is *strictly included* and we write $[R',t_k,u_\ell ,c] \prec [R,s_i,r_j,c]$.

Each such interval is associated to a minimum free energy as follows:

#### Definition 2

Let $I:=[R,s_i,r_j,c]$. $\Omega (I)$ is the set of all secondary structures that are valid for this interval, or more formally, a secondary structure *S* must fulfill:

- $$S \in \Omega (R)$$
- $s_k,r_\ell \not \in S$ for any $k<i$ and $\ell > j$
- $c=1$ implies the existance of a base pair between *s* and *r* (that is, $\{s_k,r_\ell \} \in S$ for some $i\le k \le |s|, 1 \le \ell \le j$) and $c=0$ implies that there is no such base pair.

The minimum free energy of *I* is defined as $\text {MFE}(I):= \min _{S \in \Omega (I)} E(R,S)$.

The minimum free energy of an open interval $\text {MFE}(]R,s_i,r_j,c[)$ is the minimum free energy over all secondary structures and all intervals $I' \prec I$ where *c* specifies the connectedness of *s* and *r*.

We also observe that an optimal structure is optimal for any substructure that includes all its base pairs:

#### Observation 4

If $E(R,S)=\text {MFE}([R,s_i,r_j,c])$ and *S* only contains base pairs in some interval $[R',t_k,u_\ell ,c] \preccurlyeq [R,s_i,r_j,c]$, then $S=\text {MFE}([R',t_k,u_\ell ,c])$.

We first show that our helper equation $\bar{M}$ is computed correctly:

#### Lemma 6

Assuming that $M_{R',t_k,u_\ell ,c'}=\text {MFE}(I':=[R',t_k,u\ell ,c'])$ for all $I' \preccurlyeq I:=[R,s_i,r_j,c]$, we have $\bar{M}_{R,s_i,r_j,c}=\text {MFE}(]R,s_i,r_j,c[)$.

To improve readability of the manuscript, the proof of Lemma 6 has been moved to the supplemental material.

#### Lemma 7

The algorithm computes the table entries correctly, i.e. $M_{R,s_i,r_j,c}=\text {MFE}([R,s_i,r_j,c])$ for all $R \subseteq R_0$, $s_i,r_j \in R$ and $c \in \{0,1,2\}$.

To improve readability of the manuscript, the proof of Lemma 7 has been moved to the supplemental material.

We now briefly discuss the running time. The number of table entries is bounded by $2^m \cdot n^2$, where $n:= \sum _{r \in R}|r|$ is the size of the concatenated sequence. The last case of the DP equation dominates the running time for computing one entry. In the worst case, we iterate over $2^{|R|}$ subsets and *n* entries, which gives $\mathcal {O}(2^{|R|} \cdot n)$. Partitioned by subset size, we get

$$\begin{aligned} \sum _{t=0}^m \left( {\begin{array}{c}m\\ t\end{array}}\right) n^2 \cdot 2^t n=n^3 \cdot \sum _{t=0}^m \left( {\begin{array}{c}m\\ t\end{array}}\right) 2^t= n^3 \cdot \sum _{t=0}^m \left( {\begin{array}{c}m\\ t\end{array}}\right) 1^{m-t}2^t=n^3 \cdot (1+2)^m=3^m \cdot n^3 \end{aligned}$$

which bounds the total running time. Together with lemma 7, we conclude.

#### Theorem 3

MFE Strand Interaction can be solved in time $\mathcal {O}(3^m \cdot n^3)$.

### Translation to partition function

For computing the partition function, we must take account of the arising rotational symmetries to avoid an “undercounting” of symmetrical secondary structures, before the canonical overcounting correction. We present an approach that allows to do that without iterating over all circular permutations, and can even incorporate an entropic symmetry correction as considered by [13].

We will always assume that secondary structures are connected and pseudoknot-free, and thus they have a unique pseudoknot-free permutation. In this context, we denote by $\{a,b\} \le \{c,d\}$ that base pair $\{a,b\}$ includes base pair $\{c,d\}$, that is, *c* and *d* are not outside of the interval [*a*, *b*].

**Problem of over- and undercounting of symmetries.** We introduce the notion of indistinguishability and will use the symbol $\sim$ to denote it. Two strands *s*, *t* are *indistinguishable* if their sequences are identical. Two sets $R,R'$ of strands are *indistinguishable* if there is a bijection $f:R \rightarrow R'$ such that $s \sim f(s)$ for all $s \in R$. Two pairs $((s^k)_{k \in [m]},S),((s'^k)_{k \in [m]},S')$ of a family of strands and a secondary structure are called *indistinguishable* if $s^k$ and $s'^k$ are indistinguishable for all $k \in [m]$ and $S'=\{\{s'^k_i,s'^\ell _j\} \mid \{s^k_i,s^\ell _j\} \in S\}$. An *r-symmetric* secondary structure is a secondary structure *S* with pseudoknot-free permutation $s^1,...,s^m$ such that for all $i \in [r]$,

$$\begin{aligned} ((s^k)_{k \in [m]},S) \sim ((s^{(k+i \cdot m/r) \bmod m})_{k \in [m]},S) \end{aligned}$$

In other words, there are *r* cyclic shifts that the DP algorithm will not be able to distinguish. For a secondary structure which is not *r*-symmetric for any $r>1$, our DP algorithm would count that structure *m* times (once for each “entry point” between two strands), because all cyclic shifts are distinguishable for the DP algorithm. However, in an *r*-symmetric structure, there are only *m*/*r* distinguishable entry points, and thus the secondary structure will only be counted *m*/*r* times. This danger of “undercounting” poses serious algorithmic challenges. We say that a secondary structure has *rotational symmetry* *r* if it is *r*-symmetric and that it has *maximum rotational symmetry* *r* if it has rotational symmetry *r* and for all $r'>r$, it is not $r'$-symmetric.

Let $\Omega$ be the set of all secondary structures, $\Omega _{r}$ be the set of all secondary structures with rotational symmetry *r*, and $\Omega _{\max = r}$ be the set of all secondary structures with maximum rotational symmetry *r*. We can first show a simple lemma which states that *r*-symmetric secondary structures have a multiple of *r* as their maximal rotational symmetry.

#### Lemma 8

If $S \in \Omega _r$, then $S \in \Omega _{\max = t}$ with $t \bmod r = 0$.

#### Proof

Any secondary structure has a maximum rotational symmetry. Assume for contradiction that for this maximum rotational symmetry *t* for *S*, $t \bmod r \ne 0$. We claim that *S* has rotational symmetry $s:=\text {lcm}(t,r) > t$.

First of all, we know that $m \bmod r = 0$ and $m \bmod t = 0$, since otherwise there could not be an *r*- (resp. *t*-) symmetry. Together with $m > \max (r,t)$, it follows that $m>s$ and that $m \bmod s=0$. We will assume that $m=s$, and if *m* is a multiple of *s*, we can just consider $\frac{m}{s}$ strands to be one strand.

The repeat lengths *s*/*r* and *s*/*t* are coprime and *s*/*r* has a multiplicative inverse modulo *s*/*t*, i.e. there is some *y* such that $ys/r \bmod s/t = 1$. In particular, $iy s/r \bmod s/t = i$. Consider two arbitrary strands $a^1,a^d$ in the pseudoknot-free permutation $a^1,...,a^s$. Take *y* such that $ys/r \equiv d \mod s/t$. The structures of $a^1$ and $a^{ys/r}$ have to be identical by *r*-symmetry, and by *t*-symmetry, $a^{ys/r}$ has to be identical to $a^d$. Thus the structures of all strands are identical and we have an *s*-symmetry. $\square$

Another simple observation is that a higher symmetry implies a symmetry of its divisor:

#### Observation 5

For $i,r \in \mathbb {N}^+$, if $S \in \Omega _{i \cdot r}$, then $S \in \Omega _r$.

We now define some partition function values that we want to compute:

$$\begin{aligned} \mathcal {Z}_{\max = r}&=\sum _{S \in \Omega _{\max = r}} \exp \{-E(S)/kT\}\\ \mathcal {Z}_r&= \sum _{i=1}^{\lfloor \frac{m}{r} \rfloor } \frac{m}{i \cdot r} \cdot \sum _{S \in \Omega _{\max = i \cdot r}} \exp \{-E(S)/kT\} \end{aligned}$$

Assume there is a set $R_{/r}$ of strands where each sequence appears exactly *r* times less than in *R*. There is at most one such distinguishable set, and it has size *m*/*r*. It suffices to compute a variant of the partition function over $R_{/r}$. Namely, an *extended secondary structure* is a pair of a secondary structure $S'$ and a *marked* base pair $p \in S'$. Any pair of a secondary structure and a base pair is now part of the structure space $\bar{\Omega }(R_{/r})$. As for the standard secondary structure, we can restrict the space to structures of particular symmetries, e.g. $\bar{\Omega }_{\max = i}(R_{/r})$. The free energy of an extended secondary structure is defined as $\bar{E}((S',p))= r \cdot E(S')$.

Intuitively, all predecessors of the marked base pair *p* (including itself), namely all $q \le p$, will be flipped such that the first base of the pair is moved to the next symmetrical occurrence of this base. Therefore, we will sometimes refer to marked base pairs as all $q \le p$, and not only *p* itself.

**Fig. 5:**
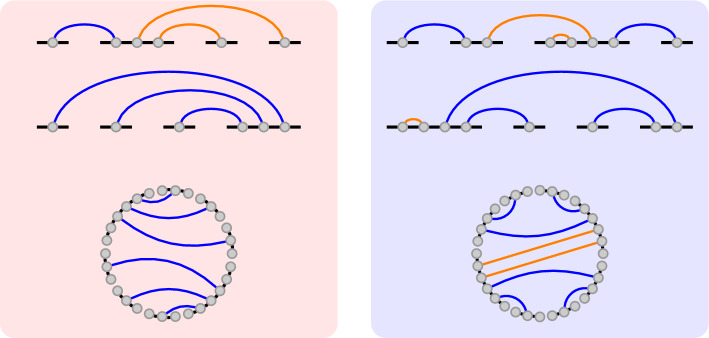
Two extended secondary structures, where marked base pairs are colored orange. On the left side (red box), the innermost marked base pair is inter-strand (top), and therefore after the cyclic shift (middle), no base pair is marked, which results in an unconnected 2-symmetric structure (bottom). On the right side (blue box), the innermost marked base pair is intra-strand (top), and therefore after the cyclic shift (middle), it is still marked. This base pair ensures the connectedness of the 2-symmetric structure (bottom)

**Cyclic shift operations on extended secondary structures.** It will be convenient to talk about *cyclic shifts* of extended secondary structures. Each strand is moved one position to the right, and the last strand is moved to the front. Additionally, if the order of the bases of a base pair changes due to the cyclic shift, we change the markedness property (including parents) of the base pair. Two examples can be found in fig. 5.

An extended secondary structure $(S',p)$ is *connected* if and only if:

- $S'$ is connected and
- *p* is interior or $S' - \{q \in S' \mid q \le p\}$ is connected.

The partition function over this space is defined as follows:

$$\begin{aligned} \bar{\mathcal {Z}}(R_{/r}) = \sum _{i=1}^{\lfloor \frac{m}{r} \rfloor } \frac{m}{i} \sum _{S \in \bar{\Omega }_{ \max = i}(R_{/r})} \exp \{-\bar{E}(S)/kT\} \end{aligned}$$

An *i*-rotational secondary structure is overcounted by a factor of $\frac{m}{i}$, which we account for in the above definition. Additionally, due to lemma 8, we only need to consider rotational symmetries which are multiples of the considered symmetry.

It is easy to extend the DP to capture this space: We add a *marked bit* *b* which is 1 if the marked base pair is in the region, and 0 else. An unpaired position does not change the marked bit. If $b=1$ and we are in the case of a single stack, we add the two values for the case when the stack base pair is marked (in that case, the inner region has $b=0$) or not. For $b=0$, we do not change anything. If $b=1$ and we are in the case of a multiloop, we add the value for the case where the first multiloop base pair is marked and the rest of the multiloop as well as the inner region has $b=0$, to the value where the marked base pair is in the inner region and the value for the case where the rest of the multiloop has $b=1$. In the end, we query the complete region with $b=1$. These extensions do not increase the asymptotic complexity of our standard DP algorithm. We can thus derive:

#### Lemma 9

$\bar{\mathcal {Z}}(R_{/r})$ can be computed in time $\mathcal {O}(3^m \cdot n^3)$.

We now describe a bijection between $\Omega _r(R)$ and $\bar{\Omega }(R_{/r})$. Fix an arbitrary strand $a \in R$. Consider $S \in \Omega _r(R)$. Relabel the strands to $s^0,...,s^{m-1}$, where $s^0=a$ and the other strands follow in the ordering of the unique pseudoknot-free permutation. Let $p_S$ be the innermost base pair in *S* such that one base is on a strand between $s^0$ and $s^{m/r-1}$, and one is not. Our function $f: \Omega _r(R) \rightarrow \bar{\Omega }(R_{/r})$ is defined as

$$\begin{aligned} f(S)=(\{\{s^b_i,t^{c \bmod \frac{m}{r}}_j\} \mid \{s_i^b,t_j^c\} \in S \wedge 0 \le b < \frac{m}{r}\}, p_S) \end{aligned}$$

#### Lemma 10

$S \in \Omega _r(R)$ is connected if and only if *f*(*S*) is connected.

#### Proof

Assume *S* is connected. Consider $f(S)=(S',p)$ and assume that $S'$ is not connected. Consider one connected component of strands in $S'$ and call it $C'$. In *S*, by its *r*-symmetry, these strands repeat in components $C_0,...,C_{r-1}$. The union of these components does not contain any outgoing base pairs, which gives a contradiction to the connectedness of *S*. Thus, $S'$ is connected.

Now assume that $S' - \{q \in S' \mid q \le p\}$ is not connected. Consider a new extended secondary structure $S''$ that is obtained by cyclic shifts of $S'$ until the strand of the second base of the innermost marked base pair is in front. Now, every marked inter-strand base pair is flipped in $S''$, and thus not marked anymore. Marked intra-strand base pairs however remain marked. If the innermost marked base pair is inter-strand, then in $S''$, no marked base pair exists, which corresponds to *r* separate connected components, a contradiction to the connectedness of *S*. Thus, the original innermost marked base pair must be intra-strand. We conclude that *f*(*S*) is connected.

For the other direction, assume $f(S)=(S',p)$ is connected. If $S'- \{q \in S' \mid q \le p\}$ is connected, each of the *r* symmetric repeats consist of at most one connected component. The marked base pair *p* then connects these connected structures with each other. Thus, *S* is connected.

If $S'- \{q \in S' \mid q \le p\}$ is not connected, there is a marked interior base pair. By connectedness of $S'$, there is a circular shift of the permutation, where marked exterior base pairs are unmarked, such that the structure without the marked base pair is connected. We can then proceed as above. $\square$

#### Lemma 11

$S \in \Omega _{r \cdot i}(R)$ if and only if $f(S) \in \Omega _{i}(R_{/r})$.

#### Proof

Assume $S \in \Omega _{r \cdot i}$. By observation 5, there are *r* symmetrical substructures and each of them is *i*-symmetric, and we thus get $f(S) \in \Omega _{i}(R_{/r})$. For the other way, $f^{-1}$ replicates the (by assumption *i*-symmetric) structure *r* times, thus the resulting structure is $r \cdot i$-symmetric. $\square$

#### Lemma 12

The function $f: \Omega _r(R) \rightarrow \bar{\Omega }(R_{/r})$ is a bijection, preserves free energy and connectedness, and decreases the rotational symmetry by a factor of $\frac{1}{r}$.

#### Proof

Connectedness follows from lemma 10, the decreasing of the rotational symmetry follows from lemma 11, and the preservation of the free energy is immediately clear since each base pair in *f*(*S*) is weighted with a factor of *r*, and in *S*, it is replicated *r* times. It thus remains to show that *f* is a bijection.

We first show injectivity. Consider two different *r*-symmetric secondary structures $S,T \in \Omega _r(R)$. Consider $f(S)=(S',p_S)$ and $f(T)=(T',p_T)$. We order the strands with respect to the correct permutation of *S*, with $r^0=a$. First we notice that if the unique pseudoknot-free permutations of *S* and *T* differ, so do the unique pseudoknot-free permutations of $S'$ and $T'$, which would imply $S' \ne T'$.

So we can assume that their pseudoknot-free permutation is the same. Because *S* and *T* are *r*-symmetric and different, there is a position $s^p_i$ for $0 \le p < \frac{m}{r}$ which is differently paired in *S* and *T*. Assume without loss of generality that $s^p_i \in S$. If $s^p_i \not \in T$, we have $s^p_i \in S'$ but $s^p_i \not \in T'$ and we are done. Else, if $s^p_i$ is matched differently in $S'$ and $T'$, we are done again. We can thus assume that $s^p_i$ is matched to the same $s^q_j$ for $0 \le q < \frac{m}{r}$ in $S'$ and $T'$. Since $s^p_i$ is differently matched in *S* and *T*, we must have $\{s^p_i,s^q_j\} \in S$ and $\{s^{p+\frac{m}{r}}_i,s^q_j\} \in T$, or the other way around. Thus, $p_T$ has to be in the region enclosed by base pair $\{s^p_i,s^q_j\}$, but $p_S$ cannot be in this region, because both endpoints of the base pair are between strands $s^0$ and $s^\frac{m}{r}$. Thus $f(S)\ne f(T)$.

We now show surjectivity. Consider an arbitrary $E \in \bar{\Omega }_r$, that is, a pair $E=(S',P)$. Now build a secondary structure *S* as follows: For easch base pair $\{s^p_i,s^q_j\}$, if it does not enclose *P*, add itself and its $r-1$ symmetrical copies to *S*. For the case that it encloses *P*, assume wlog $p<q$. We add the base pair $\{s^q_j,s^{p+\frac{m}{r}}_i\}$ and its *r* symmetrical copies to *S*. It is easy to see that $f(S)=(S',P)$.

Finally notice that the function is total, i.e. it is defined for every $S \in \Omega _r(R)$. $\square$

By lemma 12, we can now rewrite $\mathcal {Z}_r$ as follows:

$$\begin{aligned} \mathcal {Z}_r&= \sum _{i=1}^{\lfloor \frac{m}{r} \rfloor } \frac{m}{i \cdot r} \cdot \sum _{S \in \Omega _{\max = i \cdot r}(R)} \exp \{-E(S)/kT\}\\&=\sum _{i=1}^{\lfloor \frac{m}{r} \rfloor } \frac{m}{i \cdot r} \cdot \sum _{S \in \bar{\Omega }_{\max = i}(R_{/r}) }\exp \{-\bar{E}(S)/kT\}\\&=\bar{\mathcal {Z}}(R_{/r}) \end{aligned}$$

This quantity can be computed in time $\mathcal {O}(3^m \cdot n^3)$ by lemma 9. We can finally conclude:

#### Lemma 13

$\mathcal {Z}_r$ can be computed in time $\mathcal {O}(3^m \cdot n^3)$.

Using this result, we will proceed by showing that $\mathcal {Z}_{\max = r}$ can also be computed efficiently.

#### Lemma 14

$\mathcal {Z}_{\max = r}$ can be computed in time $\mathcal {O}(3^m \cdot n^3 \cdot m)$, for any *r*.

#### Proof

We can compute $\mathcal {Z}_{\max =r}(R)$ as follows. We create the following DP:

$$\begin{aligned} \mathcal {Z}[t] = \frac{t}{m} \cdot \left( \mathcal {Z}_t - \sum _{i=2}^{ \lfloor \frac{m}{t} \rfloor } \frac{m}{i\cdot t} \cdot \mathcal {Z}[i \cdot t]\right) \end{aligned}$$

Indeed, we can inductively verify the correctness of the equation, namely we can proof $\mathcal {Z}[t]=\mathcal {Z}_{ \max = t}$, with respect to the energy contribution $E'$, for all $t \in \{1,...,m\}$. For the base case $t=m$, notice that

$$\begin{aligned} \mathcal {Z}[m] = \frac{m}{m} \cdot (\mathcal {Z}_m - 0) =\mathcal {Z}_m =\sum _{S \in \Omega _{\max = m}} \exp \{-E'(S)/kT\} =\mathcal {Z}_{\max = m} \end{aligned}$$

Inductively, assume that $\mathcal {Z}[t']$ is correctly computed for all $m \ge t' > t$. We have

$$\begin{aligned} \mathcal {Z}[t]&= \frac{t}{m} \cdot \left( \mathcal {Z}_t - \sum _{i=2}^{ \lfloor \frac{m}{t} \rfloor } \frac{m}{i\cdot t} \cdot \mathcal {Z}[i \cdot t]\right) \\&=\frac{t}{m} \cdot \left( \sum _{i=1}^{\lfloor \frac{m}{t} \rfloor } \frac{m}{i \cdot t} \cdot \sum _{S \in \Omega _{\max = i \cdot t}} \exp \{-E'(S)/kT\} - \sum _{i=2}^{ \lfloor \frac{m}{t} \rfloor } \frac{m}{i\cdot t} \cdot \mathcal {Z}_{\max = i \cdot t}\right) \\&=\frac{t}{m} \cdot \left( \begin{array}{c}\displaystyle \sum _{i=1}^{\lfloor \frac{m}{t} \rfloor } \frac{m}{i \cdot t} \cdot \sum _{S \in \Omega _{\max = i \cdot t}} \exp \{-E'(S)/kT\} \\ \displaystyle - \sum _{i=2}^{ \lfloor \frac{m}{t} \rfloor } \frac{m}{i\cdot t} \cdot \sum _{S \in \Omega _{\max = i \cdot t}} \exp \{-E'(S)/kT\}\end{array}\right) \\&=\frac{t}{m} \frac{m}{t} \sum _{S \in \Omega _{\max = t}}\exp \{-E'(S)/kT\}\\&=\sum _{S \in \Omega _{\max = t}}\exp \{-E'(S)/kT\}\\&=\mathcal {Z}_{\max = t} \end{aligned}$$

By lemma 13, each $\mathcal {Z}_t$ can be computed in time $\mathcal {O}(3^m \cdot n^3)$. This dominates the running time to compute one entry. Since we compute at most *m* entries, an overall running time in $\mathcal {O}(3^m\cdot n^3\cdot m)$ follows. $\square$

Now that we have the values for $\mathcal {Z}_{\max = r}$, we can compute the value of the partition function $\mathcal {Z}$:

$$\begin{aligned} \mathcal {Z}&=\sum _{S \in \Omega } \exp \{-E(S)/kT\} =\sum _{r=1}^m \sum _{S \in \Omega _{\max = r}}\exp \{-E(S)/kT\} =\sum _{r=1}^m \mathcal {Z}_{\max = r} \end{aligned}$$

By lemma 14, we can compute each $\mathcal {Z}_{\max = r}$ in time $\mathcal {O}(3^m \cdot n^3 \cdot m)$. Since we sum over *m* such entries, we finally obtain the following result:

#### Theorem 4

The partition function $\mathcal {Z}$ over *m* strands can be computed in time $\mathcal {O}(3^m \cdot n^3 \cdot m^2)$.

#### Remark 3

It is easy to see that for each rotational symmetry *r*, we can add the symmetry correction $kT \log r$ as described by Dirks et al [13] to the DP equations, if desired. Thus, the above result also holds for this variant of the partition function.

#### Remark 4

The described technique directly translates to the other algorithms that we will present in the following sections. It can be applied to obtain the exact partition function for the triplet repeat setting (section 4.3) and the strand soup setting (section 4.5).

### Strand interactions for triplet repeats

We now consider the special case where all strands in our pool are triplet repeats. We call this restricted problem MFE Triplet Repeat Strand Interaction. Assume first that all strands have the same pattern and that we have a bounded number of different strand-lengths $p:=|\{i \mid \exists r \in R: |r|=i\}|$. Regardless of the ordering of the strands, the resulting sequence of the concatenated strands is identical. We can therefore focus on the length of the strands and disregard their actual sequence.

We do not iterate over all subsets of *R*, since we only need to distinguish the number of strands of a certain length in the subset, in a count-sort-like manner. Thus we can represent a subset $R'\subseteq R$ by $(a_1,...,a_p)$ where $a_i:=|\{r \in R' \mid |r| = n_i\}|$ is the number of strands of size $n_i$ in *R*. An example is given in Fig. 4b. As argued in the following, the exponent only depends on *p*:

We need table entries for each possible configuration of remaining number of occurrences and for specifying the remaining number of bases on the leftmost and rightmost strand. Using $n:=\max _{r \in R} |r|$, we bound the number of table entries by

$$\begin{aligned} n^2 \cdot \max _{s_1,...,s_p: s_1+...+s_p=m} \prod _{i=1}^p s_p \le n^2 \cdot \left( \frac{m}{p}\right) ^p \end{aligned}$$

The running time for computing one table entry is dominated, as for the previous section, by the last case. We need to iterate over $\mathcal {O}((\frac{m}{p})^p)$ configurations to split our region into two strand sets, *p* lengths to determine the length of the strand on which we split and *n* positions for the index of the split. We finally obtain a running time of $\mathcal {O}((\frac{m}{p})^{2p} \cdot n^3 \cdot p)$, which is an XP algorithm parametrized by *p*.

#### Theorem 5

There is an XP algorithm for MFE Triplet Repeat Strand Interaction parametrized by the number of different lengths *p*, running in $\mathcal {O}((\frac{m}{p})^{2p} \cdot n^3 \cdot p)$ time.

Notice that this algorithm can be extended to the case where we have different triplet patterns; the parameter then becomes the number of non-identical strands.

### Computational hardness

In this subsection, we show that the parametrized approach seen before is, in a sense and under standards assumptions, the best we can hope for. Moreover, even for triplet repeats, the problem of deciding whether there is a secondary structure for $R_0$ with a free energy below a certain threshold *t* remains ${{\textbf {NP}}}$-complete, for a reasonably-intricate energy model. Note that for the general (non-triplet) case, this has already been shown in [14]. Our result is surprising in the sense that the concatenation of TR strands always yields the same sequence, and the only additional difficulty compared to the single-stranded case arises from the fact that we do not know the indices of the strand borders.

Our reduction requires more than the naive base pair maximization model, but to keep the reduction simple, we will not use the full Turner energy model. Instead, we posit that each base pair gives a free energy reward of $E^\text {bp}=-\frac{m}{3}$, where $m>0$ is the number of interacting strands, while subdividing an interval into two intervals that are not strand-disjoint gives a multiloop penalty of $K_\text {multi}=+1$. Furthermore, each connected component reduces the strand association penalty by $-K_\text {assoc}:=-1$. Finally, every hairpin loop must enclose at least three unpaired bases ($\theta =3$). This model can be extended into the Turner model by setting equal energy values for interior and hairpin loops, and accounting for the multiloop penalty in the corresponding energy values.

Let us define our main decision problem: 
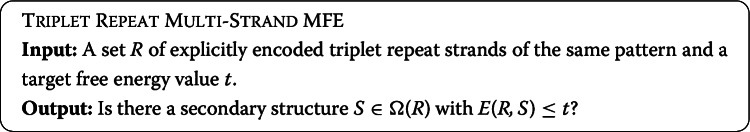
 Even if the following reduction does not work in the base pair maximization model, a DP algorithm for base pair maximization in this setting seems unlikely, as, under the assumption that ${{\textbf {P}}}\ne {{\textbf {NP}}}$, one would not be able to generalize the algorithm to more complex energy models.

We will show **NP**-hardness by reduction from the following problem: 
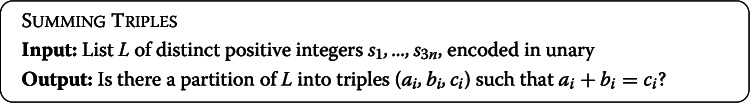


The problem was shown to be strongly NP-hard [21]. We define $v:=\sum _{i=1}^{3n}s_i$.

**Fig. 6:**
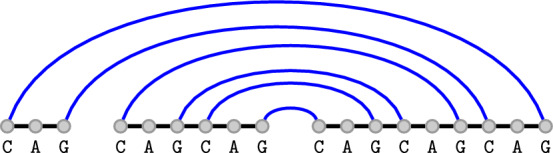
Optimal secondary structure corresponding to a valid summing triple (1, 2, 3). The $1 \cdot 3 + 2 \cdot 3$ bases on the left perfectly match to the $3 \cdot 3$ bases on the right

The reduction is as follows: We create a strand $r_i:=(CAG)^{s_i}$ for each integer $s_i$. Hence, we have $n=\frac{m}{3}=-E^\text {bp}$. We denote by *R* the set of strands. We set the target minimum free energy to $t:=-(3v+1)n$.

Assume that there is a partition into summing triples. Our secondary structure is built such that for each triple $a+b=c$, we add the base pairs

$$\begin{aligned} (a_1,c_{|c|}),(a_3,c_{|c|-2}),(a_4,c_{|c|-3}),(a_6,c_{|c|-5})&,...,(a_{|a|-2},c_{|c|-|a|+3}),(a_{|a|},c_{|c|-|a|+1}),\\ (b_1,c_{|c|-|a|}),(b_3,c_{|c|-|a|-2})&,...,(b_{|b|-2},c_3),(b_{|b|},c_1) \end{aligned}$$

Note that all base pairs are labeled with $C-G$ or $G-C$. fig. 6 visualizes the secondary structure for the exemplary triple $1+2=3$. We claim that *S* is unpseudoknotted for the circular permutation $a_1 \cdot b_1 \cdot c_1 \dots a_n \cdot b_n \cdot c_n$ and that $E(R,S)=t$.

Since any two triples of strands are not connected, we have exactly *n* connected components. Each connected component consists of one large stacked loop with innermost base pair $(b_{|b|},c_1)$ (i.e. we do not violate the constraint that every innermost base pair must include three unpaired bases, because the base pair is inter-strand). Since $a+b=c$, the outermost base pair is $(a_1,c_{|c|})$. There is no multiloop involved in *S*, so each triple $(a_i,b_i,c_i)$ contributes a free energy of $2|c| \cdot E^\text {bp} - K_\text {assoc}=-6n|c|-1$. Since all triples are correctly summing, we have $\sum _{i=1}^n c_i=\frac{1}{2} v$. Thus indeed the minimum free energy is at most

$$\begin{aligned} \sum _{i=1}^n -6n|c_i|-1 = -6n \sum _{i=1}^n |c_i| - n = -6n \cdot \frac{1}{2} v - n=-3nv-n=t \end{aligned}$$

Before showing the opposite direction, we introduce the following simple lemmata:

#### Lemma 15

If some *C* or *G* base remains unpaired in a secondary structure *S*, $E(R,S)>t$.

#### Proof

First notice that in every valid secondary structure, all *A* bases remain unpaired (since there are no *U* bases). There are 2*v* bases of *C*/*G* in total. Since we assumed that one of them is unpaired, there can be at most $v-1$ base pairs. We can have at most 3*n* complexes, so the strand association penalty is reduced by at most 3*n*. Thus we have $E(R,S)\ge -3n(v-1)-3n=-3vn>-(3v+1)n=t$. $\square$

#### Lemma 16

If *S* contains a hairpin loop, $E(R,S)>t$.

#### Proof

A hairpin loop must enclose at least three bases. Since in the ${\texttt {CAG}}$ triplet pattern any two consecutive bases involve at least one *C* or *G*, we can apply lemma 15 and conclude. $\square$

Now assume for an arbitrary $S\in \Omega$ that $E(R,S)\le t$. We first show that there must be exactly *n* connected components, each with three strands. Assume that there is a connected component with less than three strands. If it has only one strand, it must contain a hairpin loop, and by lemma 16, $E(R,S)>t$. If the complex contains two strands, first of all the two strands have a different number of triplet repeats, since all $s_i$ are distinct. This implies that if the innermost loop is inter-strand (if it is intra-strand we again apply lemma 16) and has no multiloop, some *G* or *C* base must be unpaired (since base pairs can then only be between the two strands, but one of the strands contains at least one *G* and one *C* base more than the other). Then, by lemma 15, $E(R,S)>t$. If it has a multiloop, there have to be two innermost base pairs, one of which must be intra-strand, and we can apply lemma 16.

Since we ruled out complexes of one or two strands and the total number of strand is divisible by 3, we know that if there is a complex with four strands, our secondary structure will have $<n$ connected components. Thus the best achievable score will be $-n+1-3nv>t$. Hence, any $S \in \Omega$ with $E(R,S)\le t$ consists of *n* complexes, each consisting of three strands $a_i,b_i,c_i$ with $|a_i|<|b_i|<| c_i|$. We claim that for all $i \in [n]$, $|a_i|+|b_i|=|c_i|$.

By contradiction, assume $|a_i|+|b_i|\ne |c_i|$ and first consider the case that there are no multiloops. This implies that there is only one innermost base pair. If it is intra-strand, we obtain a contradiction to $E(R,S)\le t$ by lemma 16. If it is inter-strand, all remaining base pairs must be between one of two strands *d*, *e* on the one side and the third strand *f* on the other side. Since $|d|+|e|\ne |f|$ for any such partition, one of the two sides will be left with at least one unpaired *G* and one unpaired *C*, and we apply lemma 15.

Now we consider the case of multiloops. Any multiloop where the cutpoint between the two recursive structures is on a strand border (and thus is not penalized) implies an innermost base pair in both recursive structures, and since by pigeonhole principle one of the two recursive structures is single-stranded, we have a hairpin loop and $E(R,S)>t$ by lemma 16. In the other case, we have a multiloop penalty of $+1$. Thus we can lower bound $E(R,S) \ge -n-3nv+1>t$.

This finishes the proof that $|a_i|+|b_i|=|c_i|$, and we get $\frac{|a_i|}{3}+\frac{|b_i|}{3}=\frac{|c_i|}{3}$. By the construction, each strand *r* corresponds to one integer $\frac{|r|}{3}$ in the set of integers of our original instance. Thus, $(\frac{|a_i|}{3},\frac{|b_i|}{3},\frac{|c_i|}{3})$ for all complexes $\{a_i,b_i,c_i\}$ for $1\le i \le n$ is a valid set of summing triples.

The reduction is polynomial-time, since in the Summing Triples problem, the integers are encoded in unary. Membership in ${{\textbf {NP}}}$ follows by the fact that we can evaluate the energy given a secondary structure and its unpseudoknotted circular permutation.

#### Theorem 6

Unary Triplet Repeat Multi-Strand MFE is **NP**-complete.

### Predicting *strand soup* interactions

We now consider the computational problem MFE Strand Soup Interaction as defined in section 2.2. In comparison to above, we no longer need to keep track of the (exponentially many) subsets, or consider any strand association penalty since we require one single complex, but must enforce global connectivity of the strands set. Towards this goal, we introduce a *connectivity bit* *c* such that $c=1$ indicate that the two outer strands have to be (transitively) connected in the corresponding interval, and $c=0$ if they do not need to be connected (but can still be).

**Fig. 7:**
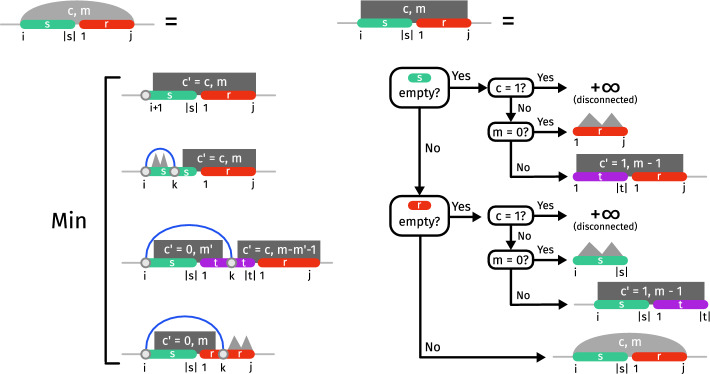
Schematic illustration of the dynamic programming scheme for the Strand Soup Interaction model (base pair-based energy model). The main case/matrix (Left) minimizes free-energy over all secondary structures for a sequence over *m* strands, beginning with a [*i*, |*s*|] suffix of the strand *s*, and ending with a prefix [1, *j*] of a strand *r*. Akin to the Nussinov recursions, the decomposition partitions the space of secondary structure based on the base-pairing status (paired/unpaired) and partner (if relevant) of the first nucleotide $s_i$, creating further base pairs through recursive calls. An auxiliary matrix (Right) uniformly handles cases where *r* or *s* may be fully depleted and one or several new strand(s) need to be inserted. For both matrices, *m* strands remain to be inserted. This number is updated and distributed over recursive calls to reflect the addition of a strand. A connectivity bit *c*, if set to true, forces the connection of *r* and *s* (either directly or transitively), ultimately ensuring that the strands end up forming a connected graph. Not shown here is the classic $\Theta (n^3)$ PD scheme to obtain the MFE of single-strands, visually indicated by *mountains* above

Let $E_{s_i,r_j}$ be the energy contribution of pairing the *i*-th base of strand *s* to the *j*-th base of strand *j*, and let $M_s[i,j]$ be the classical single-stranded minimum free energy in the interval from *i* to *j* in strand *s*. Then the minimum free-energy (BP energy model) of a secondary structure over *m* strands subject to *c*, flanked by a suffix [*i*, |*s*|] of a strand *s* and a prefix [1, *j*] of *j*, obeys:

6 $$\begin{aligned} M_{s_i,r_j,m,c}=\min {\left\{ \begin{array}{ll} \overline{M}_{s_{i+1},r_j,m,c}\\ \min _k E_{s_i,s_k} + M_s[i+1,k-1] + \overline{M}_{s_{k+1},r_j,m,c}\\ \min _{\begin{array}{c} t\in R\\ 1 \le k \le |t|\\ m'+m'' =m-1 \end{array}} E_{s_i,t_k}+\overline{M}_{s_{i+1},t_{k-1},m',0}+\overline{M}_{t_{k+1},r_j,m'',c}\\ \min _k E_{s_i,r_k}+\overline{M}_{s_{i+1},r_{k-1},m,0}+M_r[k+1,j] \end{array}\right. } \end{aligned}$$

with the following auxiliary table, responsible for the introduction of new strands whenever those identified by *s* and *r* have been entirely *consumed*:

7 $$\begin{aligned}  &   \overline{M}_{s_i,r_j,m,c}={\left\{ \begin{array}{ll} \left. {\left\{ \begin{array}{ll} \min _{t \in R} \overline{M}_{t_1,r_j,m-1,1} &  \text {if }c=0\text { and }m>0 \\ M_r[1,j] &  \text {if }c=0\text { and }m=0 \\ \infty &  \text {if }c=1 \end{array}\right. }\right| & \text {if }i> |s| \\ \left. {\left\{ \begin{array}{ll} \min _{t\in R} \overline{M}_{s_i,t_{|t|},m-1,1} &  \text {if }c=0\text { and }m>0 \\ M_s[i,|s|] &  \text {if }c=0\text { and }m=0 \\ \infty &  \text {if }c=1 \end{array}\right. }\right| &  \text {else if }j < 1 \\ M_{s_i,r_j,m,c} &  \text {otherwise} \end{array}\right. } \end{aligned}$$

The minimum free energy can be finally computed by

8 $$\begin{aligned} E^*(R,m)=\min _{s,r \in R} M_{m-2,s_1,r_{|r|},1} \end{aligned}$$

and the optimal secondary structure can be obtained through backtracking. We initialize $M_{1,s_i,s_j,2}=0$ for all $j-i\le \theta$. Equation 6 is schematically depicted in Fig. 7 (left) together with its auxilary table (Eq. 7) depicted on the right of Fig. 7.

#### Theorem 7

For any strand set *R* and number *m* of strands, the value found in $E^*(R,m)$, following its computation through Equations (6), (7) and (8), is the MFE of a connected secondary structure over *m* strands.

To improve readability of the manuscript, the proof of correctness has been moved to the supplemental material.

#### Complexity

Regarding the running time the number of entries, needed to compute the DP tables in Equations (6) and (7), is bounded by $\mathcal {O}(m \cdot p^2 \cdot n^2)$ entries, with $n:= \max _{s \in R} |s|$. The running time to compute one table entry is dominated by the repeated evaluation of the third line in Equation (6), where we minimize over $\mathcal {O}(m \cdot p \cdot n)$ different ways to introduce a new strand *t*, figure out a base pairing partner $k\in [1,|t|]$ for $s_i$, and split *m* into $(m',m'')$ such that $m'+m'' = m-1$. In total, we obtain an algorithm with running time $\mathcal {O}(n^3 \cdot m^2 \cdot p^3)$. We can then conclude:

##### Theorem 8

MFE Strand Soup Interaction over *m* strands, taken from a collection of *p* sequences, can be solved in time $\mathcal {O}(n^3\cdot m^2 \cdot p^3)$.

##### Remark 5

In addition to restricting the number of interacting strands, one can extend the above algorithm to restrict the size of the concatenated sequence. This is possible by keeping track of the current size of the sub-interval in the DP tables, and updating these values whenever a new strand is introduced. This might be useful if the sequences in the base set have different length, as the basic algorithm would otherwise favor larger sequences because they usually allow for more base pairs.

##### Remark 6

The case of triplet repeats induces a slight improvement of the running time. Since all strands look the same except for their length, we can use a table with entries of the form $M_{m,i,j,c}$, where *i* and *j* denote the remaining number of bases in the leftmost and rightmost strand. This reduces the space complexity to $\mathcal {O}(m \cdot n^2)$, but the computation of one table entry still requires the same time, giving an overall time complexity of $\mathcal {O}(n^3 \cdot m^2 \cdot p)$.

#### Incorporating a simple nearest-neighbor energy model

The DP scheme underlying Equations (6), (7) and (8) can be modified to capture a simplified nearest neighbor model, where the energy of a secondary structure *S* over *m* strands. It crucially requires the definition of an *interaction loop* between two strands *s* and *r* delimited by two base pairs $(s_i,r_j)$ and $(s_{i'},r_{j'})$ such that $1\le i<i'\le |s|$ and $1\le j'<j \le |r|$. We denote by $\Delta G (s_i,s_i',r_j',r_j)$ the energy of an interaction loop $(s_i,s_i',r_j',r_j)$, accessible via table lookup in popular libraries such as the Vienna package [22]. We can then define the simplified Turner energy of a secondary structure *S* as:

$$\begin{aligned} E(S;s_1,\ldots ,s_m) = \sum _{\text {strand }s_i} E^{\mathcal {T}}( S|_{s_i}; s_i) + \sum _{\begin{array}{c} {Interaction loop } (s_i,s_i',r_j',r_j)\\ \text {(stack, bulge, or interior loop)} \end{array}} \Delta G (s_i,s_i',r_j',r_j)\end{aligned}$$

where $S|_{s_i}$ represents the restriction of the (multi-strand) secondary structure *S* to closed substructures occurring within the strand *s*.

**Fig. 8:**
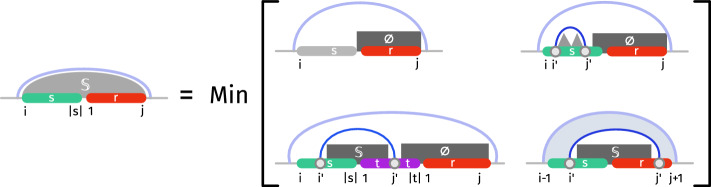
Partial illustration of our extended DP scheme, capturing a simple Turner-style energy model in the strand soup paradigm. The decomposition for filling the $M^\mathbb {S}$ matrix postulates the existence of an enclosing base pair $(i-1,j+1)$, and investigates the first paired position $i'\ge i$ in the [*i*, |*s*|] region of *s*: Such a position may not exist (*s*[*i*, |*s*|] fully unpaired), or be paired to $j''$ located in *s*, in a new strand *t*, or in *r*. In the latter case, the existence of two consecutive nested base pairs between *s* and *r* represents an interaction loop (stacked pairs, bulges or interior loop), usually associated with an energy bonus

The MFE in such a model can be obtained through a minor update of the dynamic programming scheme described in the previous section and depicted in Fig. 8, where:

- Individual base pairs no longer contribute to the free-energy individually, yet still contribute as constitutive of loops;
- Since interaction loops are characterized by a pair of directly nested base pairs, each involving two strands, we duplicate both matrices *M* and $\overline{M}$ to indicate the presence ($M^{\mathbb {S}}$ and $\overline{M}^{\mathbb {S}}$) or absence ($M^{\varnothing }$ and $\overline{M}^{\varnothing }$) of an enclosing base pair $(s_{i-1},r_{j+1})$;
- The rules of $M^{\mathbb {S}}$ need to be adapted to explicitly make the first base pair $(i',j')$ such that $i\le i'$ and $j'\le j$, available for scoring. If no such base pair exist, then *s* needs to be entirely consumed, and a new strand be allocated by subsequent calls;
- $\overline{M}^{\mathbb {S}}$ and $\overline{M}^{\varnothing }$ essentially act as above, but also respectively propagate the presence/absence of an enclosing base pair;
- The MFE contribution of a substructure formed within a region of a strand *s* is now denoted as $M^{\mathbb {S}}_s[i,j]$ if enclosed by a base pair $(s_i,s_j)$, or $M^{\varnothing }_s[i,j]$ otherwise. In the Turner energy model, those values are classically computed in time $\mathcal {O}(|s|^4)$, e.g. using the Zuker/Stiegler DP scheme [8].

9 $$\begin{aligned} M^{\varnothing }_{s_i,r_j,m,c}&=\min {\left\{ \begin{array}{ll} \overline{M}^{\varnothing }_{s_{i+1},r_j,m,c}\\ \min _{i \le k \le |s|} M^\mathbb {S}_s[i,k] + \overline{M}^{\varnothing }_{s_{k+1},r_j,m,c}\\ \min _{\begin{array}{c} t\in R\\ 1 \le k \le |t|\\ 0 \le m' < m \end{array}} \overline{M}^{\mathbb {S}}_{s_{i+1},t_{k-1},m',0}+\overline{M}^{\varnothing }_{t_{k+1},r_j,m-m'-1,c}\\ \min _{1 \le k \le j} \overline{M}^{\mathbb {S}}_{s_{i+1},r_{k-1},m,0}+M^{\varnothing }_r[k+1,j] \end{array}\right. }\end{aligned}$$

10 $$\begin{aligned} M^{\mathbb {S}}_{s_i,r_j,m,c}&=\min {\left\{ \begin{array}{ll} \overline{M}^{\varnothing }_{s_{|s|+1},r_j,m,c}\\ \min _{i\le i'< k \le |s|} M^\mathbb {S}_s[i',k] + \overline{M}_{s_{k+1},r_j,m,c}\\ \min _{\begin{array}{c} t\in R\\ i \le i' \le |s|, 1 \le k \le |t|\\ 0 \le m'< m \end{array}} \overline{M}^{\mathbb {S}}_{s_{i'+1},t_{k-1},m',0}+\overline{M}^{\varnothing }_{t_{k+1},r_j,m-m'-1,c}\\ \min _{\begin{array}{c} i \le i' \le |s|\\ 1 \le k \le j \end{array}} \Delta G(s_{i-1},s_{i'},r_{k},r_{j+1})+\overline{M}^{\mathbb {S}}_{s_{i'+1},r_{k-1},m,0} \end{array}\right. } \end{aligned}$$

11 $$\begin{aligned} \overline{M}^{\xi \in \{\varnothing ,\mathbb {S}\}}_{s_i,r_j,m,c}&={\left\{ \begin{array}{ll} \left. {\left\{ \begin{array}{ll} \min _{t \in R} \overline{M}^{\xi }_{t_1,r_j,m-1,1} &  \text {if }c=0\text { and }m>0 \\ M^{\xi }_r[1,j] &  \text {if }c=0\text { and }m=0 \\ \infty &  \text {if }c=1 \end{array}\right. }\right| & \text {if }i> |s| \\ \left. {\left\{ \begin{array}{ll} \min _{t\in R} \overline{M}^{\xi }_{s_i,t_{|t|},m-1,1} &  \text {if }c=0\text { and }m>0 \\ M^{\xi }_s[i,|s|] &  \text {if }c=0\text { and }m=0 \\ \infty &  \text {if }c=1 \end{array}\right. }\right| &  \text {else if }j < 1 \\ M^{\xi }_{s_i,r_j,m,c} &  \text {otherwise} \end{array}\right. }\end{aligned}$$

12 $$\begin{aligned} E^*(R,m)&=\min _{s,r \in R} M^{\varnothing }_{m-2,s_1,r_{|r|},1} \end{aligned}$$

The complexity of this algorithm is increased to $\mathcal {O}(n^4\cdot m^2\cdot p^3)$ with $n:= \max _{s \in R} |s|$.

## Empirical studies

The goal of this section is to show how the algorithms described in the previous section can be used to answer biologically relevant questions regarding triplet repeats. We implemented the algorithm described in section 4.5, which hereunder we call SoupFold, as well as its partition function equivalent, together with a (stochastic) backtracking procedure. The source code to reproduce our analyses is available at:

https://github.com/kimonboehmer/soupfold/

**Fig. 9:**
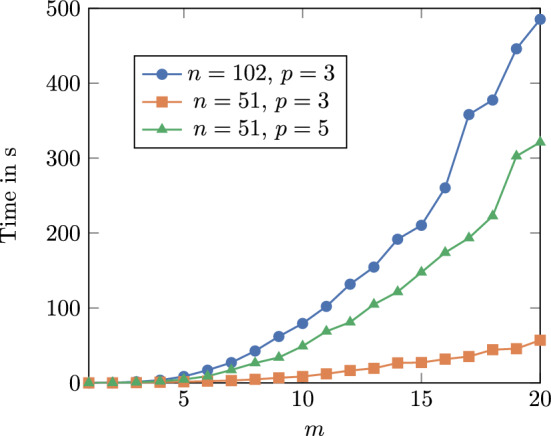
Empirical running time for increasing *m* and various values for *n* and *p*

Since we only limit the number of interacting strands but not their size, without further restrictions, the program would prefer large strands since they usually give more base pairs. To counteract this effect, we introduce a penalty on the length of a strand. Note that one could also set a maximum length of the concatenated sequence, as described in remark 5. The empirically observed running time matches the theoretical running time well, as can be seen in Fig. 9.

Regarding the stochastic backtracking, we must account for the overcounting of rotationally asymmetric secondary structures as well as for the overcounting because of the positioning of different connected components. We address these two issues by rejection sampling.

In theory, it would also be necessary to adjust the overcounting correction for rotationally symmetric structures (because they are overcounted less often) but our experiments showed that the observed probability of encountering such rotational symmetries is 0 for triplets with 15 repeats or more. Thus, for efficiency reasons, we do not include this case in our rejection sampling, arguing that the changes to the probability would be too small to observe.

**Fig. 10:**
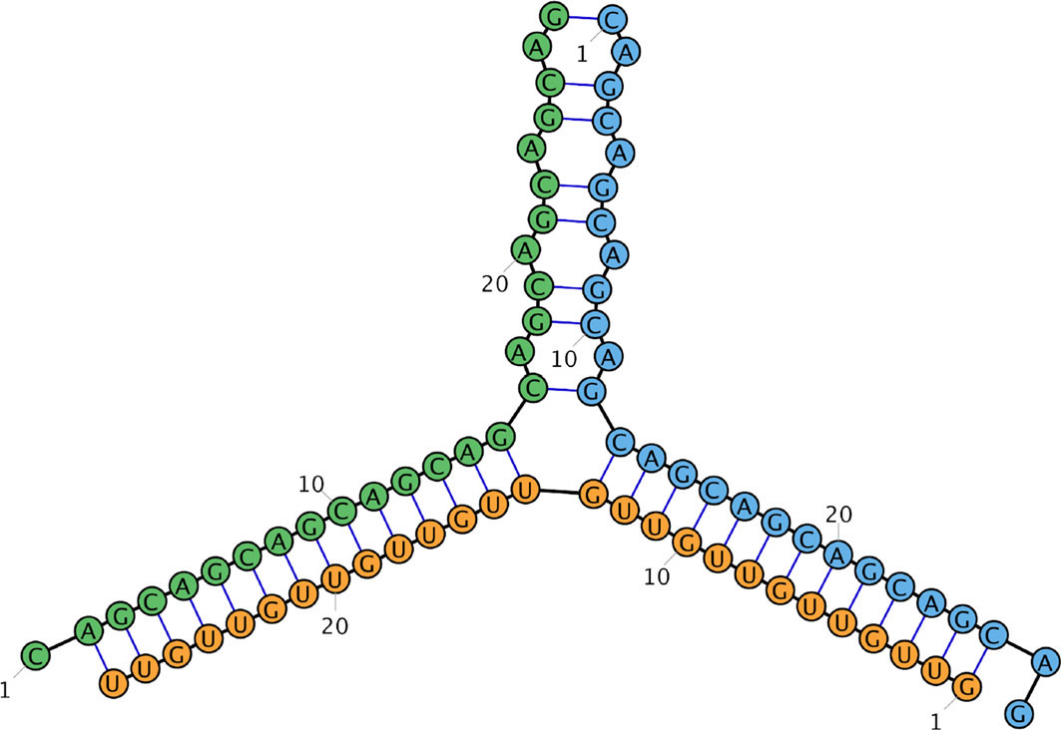
Exemplary MFE structure for strand pool $\{({\texttt {GUU}})^9,({\texttt {CAG}})^9,({\texttt {ACG}})^9\}$ computed by SoupFold with $m=3$ (RiboSketch [23])

### Homogeneous triplet soup

We first consider the case where all strands are of the same pattern.

The MFE of a soup of homogenous triplets behaves canonically, in the sense that all folding patterns have almost identical MFE structures (as can be expected, considering our results on single-strand TR in section 3). Furthermore, we observed that the number of base pairs increases canonically with the sequence length and with the number of interacting strands (except for the case of only one strand, where we loose one base pair due to a hairpin loop).

**Fig. 11:**
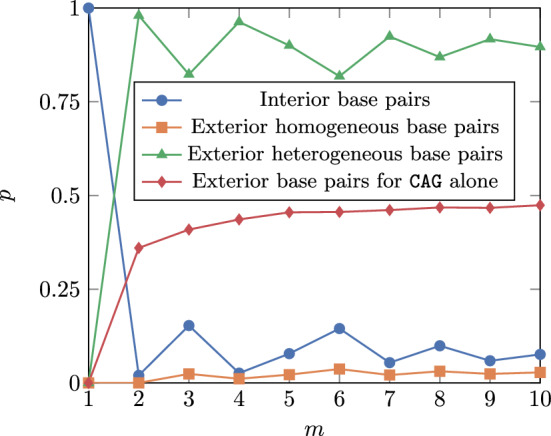
Probability *p* that a certain type of base pair is observed for increasing #strands *m*, either in a soup $\{\texttt {CAU}_{20},\texttt {GGG}_{20}\}$, or for $\texttt {CAG}_{20}$

**Fig. 12:**
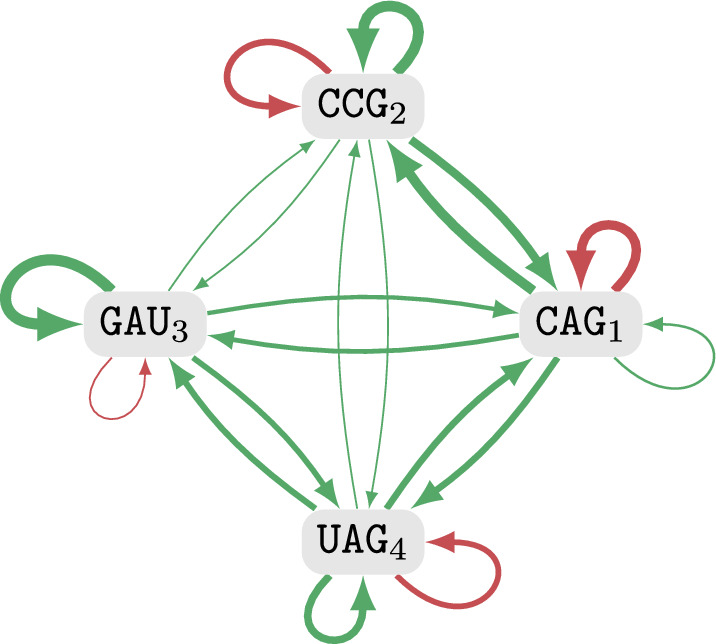
Affinity of triplet repeats (%BPs in soup model, coded by line thickness) for external (green) and internal (red) interactions

### Heterogeneous triplet soup

More interesting observations can be made in a heterogeneous pool. We can observe that different TR pattern strands can achieve more base pairs than the theoretical upper bound for a homogeneous strand pool (see Fig. 10).

In order to assess the capability of different strand soups to form droplets, we want to determine the probability of a base pair in the Boltzmann ensemble being between two strands (*exterior)* as opposed to folding (*interior*). If the strand soup consists only of triplets of one pattern, all exterior base pairs will be *homogeneous*, as opposed to *heterogeneous* for an interaction of two strands of different patterns. In the homogeneous case, we can observe an increase of exterior base pairs for increasing number of interacting strands *m*, as presented by the red line in Fig. 11. The probabilities in a setting with strands of different patterns are much richer and less canonical, as can be seen at the example of the interaction of CAU and GGG, presented by the other lines in Fig. 11. These probabilities highly depend on the number of strands, and only start to “converge” with quite high values of *m*.

**Fig. 13:**
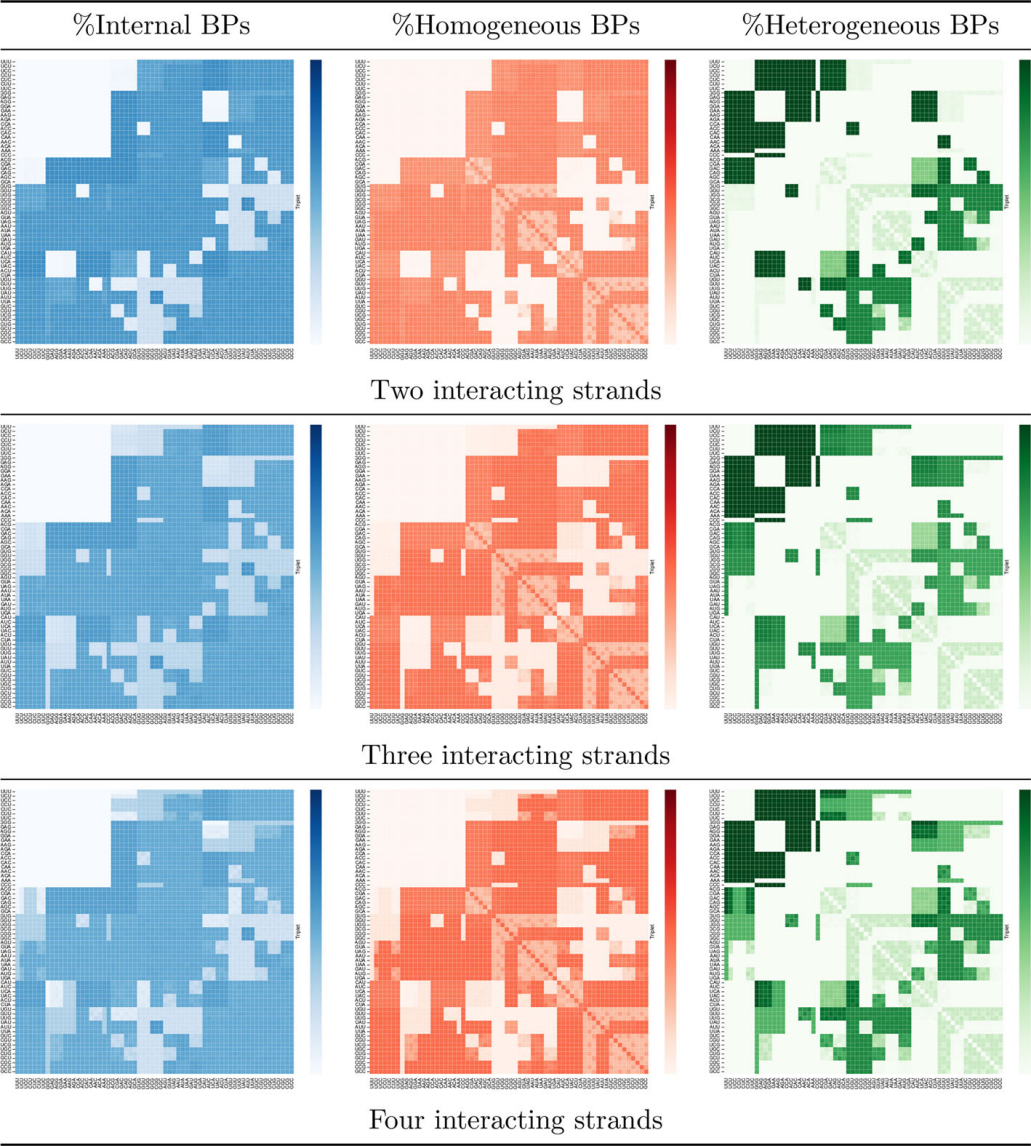
Interaction profiles for pairs of triplets in the heterogeneous soup model

To obtain a broader picture, we performed stochastic backtracking on all possible $4^6$ pairs of triplet repeat patterns $\{TVW,XYZ\}$ as strand sets, with *m* between 2 and 5, and computed the probability of a base pair being interior, exterior-homogeneous or exterior-heterogeneous. Figure 13 shows the probability of interior, exterior-homogeneous and exterior-heterogeneous base pairs for all pairs of TR, from $m=2$ to 4. We can observe that the probabilities vary a lot and highly depend on the interacting triplets. Some pairs of triplets do not form base pairs at all, in which case all three corresponding tables have a blank entry. Usually, internal and exterior-homogeneous base pairs behave similarly. One can also see that the probability of heterogeneous base pairs slightly increases with increasing *m*. On the other hand, the probability of observing interior base pairs is slightly decreasing.

From a synthetic biology perspective, some triplet repeats aggregate and form a Liquid-Liquid Phase Separation, which can be used to isolate subprocesses, thereby implementing a notion of orthogonality. In order to maximize the number of independent tasks being performed by a modified bacteria, it would then be desirable to find a large number of triplet repeat patterns such that the probability of heterogeneous base pairs is low.

For that, we can model the patterns as vertices of a graph and draw an edge if the heterogeneous base pair probability between two patterns for $m=5$ is high (we set the threshold to 0.175). We then want to determine a maximum independent set (MIS), i.e. the largest set of triplets that do not have a high probability of interacting pairwise with each other. We used an exact solver [24] to obtain a MIS of size 4, namely $\texttt {CAG, CCG, GAU, UAG}$.

We then executed our algorithm on these triplet patterns as strand soup, and could indeed observe that the probability of exterior heterogeneous base pairs is clearly below 0.2 for values of *m* between 1 and 10. In Fig. 12, we depict the number of base pairs that are between two types of strands, for $m=5$ and our four independent TR patterns as strand soup. We added a bonus to the appearance of strands, to ensure that all strands of the soup appear equally often in the constructed structures. We observe that for three of the four triplets, for exterior base pairs, the most likely interacting strand is of the same type.

## Conclusion and discussion

In this work, we investigated the algorithmic aspects of folding and interactions of triplet repeat RNA sequences, while also revisiting the general (non-triplet) setting in the interaction setting. For the folding of individual triplets, we found that their repetitive structure allows us to immediately characterize the MFE and partition function value, without the need of a more time-consuming DP approach. For interactions of RNA sequences, we exhibited a new algorithm with improved running time that avoids the factorial-time iteration over all permutations and acts as a foundation for the design of specialized algorithms, as the XP algorithm for triplet repeats. For the “strand soup” setting, we derived a polynomial-time algorithm and demonstrated possible uses for experiments regarding triplet repeats.

For future work, it is desirable to describe in detail how to extend the MFE Strand Interaction algorithm to the full thermodynamic setting considered in [13]. While the extension to the Turner model does not pose any algorithmic challenges, it would be interesting to implement a variant of the inside/outside algorithm to compute exactly base-pairing probabilities and other expected values of additive properties. Finally, the joint conformation space explored in this work is heavily restricted by the existence of a non-crossing strand ordering. More complex conformational spaces could be captured by using DP approaches akin to those used to include pseudoknots in RNA structure prediction.

## Additional file

Supplementary file 1 (pdf 308 KB)

## Data Availability

No datasets were generated or analysed during the current study.

## Declarations

### Code availability

https://github.com/kimonboehmer/soupfold/
