# Supplementary material for: RNA triplet repeats: improved algorithms for structure prediction and interactions
Source: Algorithms Mol Biol. 2025 Dec 10;21:4. doi: 10.1186/s13015-025-00292-8 (PMC13014723; doi:10.1186/s13015-025-00292-8)
Supplement: Supplementary file 1 — (pdf 308 KB) [file 13015_2025_292_MOESM1_ESM.pdf]

Supplemental material

RNA Triplet Repeats: Improved Algorithms for Structure Prediction and Interactions

Kimon Boehmer[1], Sarah J. Berkemer[1,2], Sebastian Will[1], Yann Ponty[1*]

[1*]Laboratoire d'Informatique de l'Ecole Polytechnique (LIX CNRS UMR 7161), France, Institut Polytechnique de Paris, 1 Rue Honoré d'Estienne d'Orves, Palaiseau, 91120, France.

[2]Earth-Life Science Institute, Institute of Science Tokyo, 2-12-1-I7E-318 Ookayama, Tokyo, 152-8550, Japan.

*Corresponding author(s). E-mail(s): yann.ponty@lix.polytechnique.fr; Contributing authors: kimon.bohmer@ens-paris-saclay.fr; berkemer@lix.polytechnique.fr; sebastian.will@polytechnique.edu;

Abstract

RNAs composed of Triplet Repeats (TR) have recently attracted much attention in the field of synthetic biology. We study the mimimum free energy (MFE) secondary structures of such RNAs and give improved algorithms to compute the MFE and the partition function. Furthermore, we study the interaction of multiple RNAs and design a new algorithm for computing MFE and partition function for RNA-RNA interactions, improving the previously known factorial running time to exponential. In the case of TR, we show computational hardness but still obtain a parameterized algorithm. Finally, we propose a polynomial-time algorithm for computing interactions from a base set of RNA strands and conduct experiments on the interaction of TR based on this algorithm. For instance, we study the probability that a base pair is formed between two strands with the same triplet pattern, allowing an assessment of a notion of orthogonality between TR.

Keywords: RNA folding, RNA interactions, triplet repeats, dynamic programming, NP-hardness

Preliminary Remarks

Section numbering as well as numbering of observations, lemmata and theorems are consistent with the corresponding elements in the main manuscript. As we moved the long proofs to the supplement, the corresponding numbers hopefully facilitate the search from the lemma to the matching proof.

3 Single-Stranded Triplet Repeats

Observation 2. *A secondary structure S for $(XYZ)^k$ fulfills minimum base pair span θ with $\theta \equiv_3 2$ if and only if it fulfills minimum base pair span $\theta + 1$.*

Lemma 1. *Consider a TR sequence $s := (XYZ)^k$ and a minimum number of enclosed bases $\theta \geq 0$, such that $\lfloor \frac{\theta+1}{3} \rfloor \leq k$. We have $E_{bp}(s, S) \leq k - \lfloor \frac{\theta+1}{3} \rfloor$ for any $S \in \Omega(s)$.*

Lemma 2. *For $\theta \in \{0, 1\}$ and $k > 1$, we always have $E(s, S) = k$ for any secondary structure S over a folding sequence $s = (XYZ)^k$.*

Lemma 3. *Let $\theta > 1$. The minimum MFE structure of a folding sequence $(XYZ)^k$ has value*

- $k - 1 - \frac{\theta-1}{3}$, *if* $(\{X, Z\} \notin P \wedge (\theta + 3k) \equiv_6 4) \vee (\{X, Y\}, \{Y, Z\} \notin P \wedge (\theta + 3k) \equiv_6 1)$
- $k - \lfloor \frac{\theta+1}{3} \rfloor$, *otherwise*

Furthermore, a minimum MFE structure is obtained by a single helix of base pairs of one letter pair p. If both $\{X, Z\} \in P$ and one of $\{X, Y\}$ and $\{Y, Z\} \in P$, we set $p := \{X, Z\}$ if $(\theta + 3k) \equiv_6 4$ and $p := \{X, Y\}$ (or $p := \{Y, Z\}$) if $(\theta + 3k) \equiv_6 1$; otherwise, we set p to the letters of an arbitrary pairable base pair.

Proof. We start by showing that the corresponding secondary structures achieve the claimed score. By observation 2, we only need to consider $\theta \equiv_3 0$ and $\theta \equiv_3 1$.

First assume $\{X, Z\} \in P$ and $\{X, Y\}, \{Y, Z\} \notin P$. We will derive the other cases from this one. Consider a large stacking of $X - Z$ bases. If $\theta = 3$, we only cannot match the $X - Z$ pair of the innermost repeat in the case $k \equiv_2 1$ and we only cannot match the $Z - X$ pair between the two innermost repeats in the case $k \equiv_2 0$. For all other pairs of repeats we obtain exactly two base pairs and hence we get $k - 1 = k - \lfloor \frac{\theta+1}{3} \rfloor$ base pairs. Inductively, let us show that we can obtain $k - \lfloor \frac{\theta'+1}{3} \rfloor$ base pairs for $\theta' := \theta + 3$. In other words, we only need to show that by increasing θ by 3, we get one base pair less. If the innermost base pair is $X - Z$, its enclosed region starts and ends with a Y and there are currently at least $\theta + 1$ free enclosed bases (because the region is of the form $Y(ZXY)^{\theta/3}$), and by deleting the $X - Z$ base pair, we obtain $XY(ZXY)^{\theta/3}Z$, that is $\theta + 3$ enclosed bases. Else, for a $Z - X$ base pair, the region has the form $(XYZ)^{\theta/3}$. After deleting the innermost base pair $Z - X$, the new enclosed region starts and ends with a Y (the region is of the form $YZ(XYZ)^{\theta/3}XY$), so there are at least $\theta + 4$ enclosed bases. Thus we can achieve $k - \lfloor \frac{\theta+1}{3} \rfloor$ base pairs.

If $\theta \equiv_3 1$, we distinguish two equivalence classes: In the first, k is even and $\theta \equiv_6 1$ *or* k is uneven and $\theta \equiv_6 4$, and in the second equivalence class, we have the other two cases.

For $\theta = 4$, for $k \equiv_2 1$, our lemma only claims $k-2$ base pairs. We can indeed leave the innermost repeat as well as the next $Z - X$ pair unpaired, and greedily create stackings outside of this region, obtaining $k-2$ base pairs. For $k \equiv_2 0$, We can proceed as for the even case in $\theta = 3$.

Consider $\theta + 3$ now. We add an unpaired triplet in the middle of the sequence. Now, the number of base pairs is equal to the case $k - 1$ (of opposite parity) with θ enclosed bases.

We thus established the lower bound for the $\{X, Z\} \in P$ case. For the "otherwise"-case, lemma 1 already gives us the required upper bound. Therefore, we only need to argue about the upper bound $k - 1 - \frac{\theta-1}{3}$ in the case that $\{X, Y\}, \{Y, Z\} \notin P$ and $(\theta + 3k) \equiv_6 1$. Assume a secondary structure that achieves more base pairs. Firstly, we cannot have any multiloops or exterior loops since that would imply two regions of unpaired enclosed bases, which then only allows $k - 2\lfloor \frac{\theta+1}{3} \rfloor \leq k - 1 - \frac{\theta-1}{3}$ base pairs. Additionally, for each secondary structure S with $i < j'$ and $k > 0$ such that $\{i, j'\} \in S$ and the interval $[j' + 1, j' + 3k]$ only consists of unpaired bases, we can delete the base pair $\{i, j'\}$ and instead add base pair $\{i, j' + 3k\}$ without reducing the number of base pairs. In other words, for any interval, it is always better to pair the leftmost base to the rightmost possible base than to any other interior base. We thus only need to consider the canonical structures of $X - Z/Z - X$-stackings.

Consider an odd k with all base pairs in the canonical way (for $\theta = 4$). The innermost triplet repeat bases X and Z have to stay unpaired, as well as the Z and X which are adjacent to that repeat. The innermost base pair $X - Z$ now has $7 = \theta + 3$ enclosed bases. We thus have $k - 2$ base pairs. Inductively, for $\theta' := \theta + 6$, the next two innermost base pairs will have $\theta + 3 < \theta'$ and $\theta + 3 + 2 < \theta'$ enclosed bases, thus are both not available.

Consider an even k with all base pairs in the canonical way (for $\theta = 7$). The two innermost triplet repeats have to stay unpaired, as well as the Z and X which are adjacent to that repeat. The innermost base pair $X - Z$ now has $10 = \theta + 3$ enclosed bases. The rest of the argument is exactly as above.

If $\{X, Z\} \notin P$, we can assume without loss of generality that $\{X, Y\} \in P$ (the arguments are symmetrical for $\{Y, Z\} \in P$, and we assumed to have a folding strand). We can reduce any such instance $(XYZ)^k$ to $(YZX)^{k-1}$ (by letting out the leftmost X and the rightmost Y and Z, and implicitly pairing these outermost X and Y, which is always optimal). Thus, all results can be directly obtained from the case $\{X, Z\} \in P$, by changing odd and even. The upper bound can also be derived by that. □

4 Interaction of Triplet Repeats

We also observe that an optimal structure is optimal for any substructure that includes all its base pairs:

Observation 4. *If $E(R, S) = MFE([R, s_i, r_j, c])$ and S only contains base pairs in some interval $[R', t_k, u_\ell, c] \preccurlyeq [R, s_i, r_j, c]$, then $S = MFE([R', t_k, u_\ell, c])$.*

We first show that our helper equation $\bar{M}$ is computed correctly:

Lemma 6. *Assuming that* $M_{R',t_k,u_\ell,c'} = MFE(I' := [R', t_k, u\ell, c'])$ *for all* $I' \preccurlyeq I := [R, s_i, r_j, c]$*, we have* $\bar{M}_{R,s_i,r_j,c} = MFE(]R, s_i, r_j, c[)$*.*

Proof. We distinguish four cases:

- **Case 1:** $i+1 \leq |s|$ and $j-1 \geq 1$. In that case, for any $I' \prec I$, we have $I' \preccurlyeq [R, s_{i+1}, r_{j-1}, c]$ and thus $\text{MFE}(I') \geq \text{MFE}([R, s_{i+1}, r_{j-1}, c]) = \bar{M}_{R,s_i,r_j,c}$ by assumption. Thus $\text{MFE}(]R, s_i, r_j, c[) = \bar{M}_{R,s_i,r_j,c}$.
- **Case 2:** $i+1 > |s|$ and $j-1 \geq 1$. For any $I' \prec I$, there is a $t \in R - \{s\}$ and a $c' \in \{0,1\}$ with $I' \preccurlyeq [R - \{s\}, t_1, r_{j-1}, c']$. It thus suffices to minimize over the strands $R - \{s, r\}$ while taking into account a possible strand disconnection reward. We have $\min_{t \in R-\{s,r\}, c' \in \{0,1\}} M_{R-\{s\},t_1,r_{j-1},c'} - \mathbb{1}_{c'=0} K_{\text{assoc}} = \text{MFE}(]R, s_i, r_j, c[)$.
- **Case 3:** $i+1 \leq |s|$ and $j-1 < 1$. This case is completely symmetrical to Case 2.
- **Case 4:** $i+1 > |s|$ and $j-1 < 1$. For any $I' \prec I$, there are $t, u \in R - \{s, r\}$ with $I' \preccurlyeq [R - \{s, r\}, t_1, u_{|u|}, 2]$. It thus suffices to minimize twice over the strands $R - \{s, r\}$ while taking into account a possible strand disconnection reward. We have $\min_{t,u \in R-\{s,r\}, c' \in \{0,1\}} M_{R-\{s,r\},t_1,u_{|u|},c'} - \mathbb{1}_{c'=0} K_{\text{assoc}} = \text{MFE}(]R, s_i, r_j, c[)$.

□

Lemma 7. *The algorithm computes the table entries correctly, i.e.* $M_{R,s_i,r_j,c} = MFE([R, s_i, r_j, c])$ *for all* $R \subseteq R_0$*,* $s_i, r_j \in R$ *and* $c \in \{0, 1, 2\}$*.*

Proof. We proceed by induction over the well-founded relation $\preccurlyeq$. Regarding the initialization, clearly no base pair can exist over an empty strand set, as well as over one strand where the number of enclosed base pairs between i and j is less than θ. Therefore, these table entries are correctly initialized by 0.

Let us assume that all $M_{R',t_k,u_\ell,c}$ with $[R', t_k, u_\ell, c] \preccurlyeq [R, s_i, r_j, c]$ except $M_{R,s_i,r_j,c}$ itself have been computed correctly.

- **Case 1:** $s_i \notin S$. If $i+1 \leq |s|$, we have $E(R, S) = \text{MFE}([R, s_{i+1}, r_j, c]) = M_{R,s_{i+1},r_j,c}$ by observation 4 and our induction hypothesis.
  Else, we first assume $c \neq 1$. Consider the strand t that follows s in the polymer graph of S and consider the value c' that specifies connectivity between t and r in S. Since i is unpaired, we again have $E(R, S) = \text{MFE}([R - \{s\}, t_1, r_j, c']) - \mathbb{1}_{c'=0} K_{\text{assoc}} = M_{R-\{s\},t_1,r_j,c} - \mathbb{1}_{c'=0} K_{\text{assoc}}$ as above.
  Finally, if $c = 1$, we look for the MFE of a structure in $[R, s_i, r_j, c]$ where s and r are connected by a base pair. Since there is only one base in s remaining and we leave it unpaired, there is no such structure and thus $\text{MFE}([R, s_i, r_j, 1]) = +\infty$.
- **Case 2:** $S = \{\{s_i, r_j\}\} \cup S'$, where S' is the best structure for any $I' \prec [R, s_i, r_j, c]$ with s and r arbitrarily connected (that is, $]R, s_i, r_j, 2[$). First assume $c \neq 0$. In this case, we have $E(R, S) = E_{s_i,r_j} + \text{MFE}(]R, s_i, r_j, 2[) = E_{s_i,r_j} + \bar{M}_{R,s_i,r_j,2}$, where we could apply lemma 6 because of the induction hypothesis.
  Now assume $c = 0$. We minimize over all structures such that s and r are not connected, but require $\{s_i, r_j\} \in S$. Thus $\text{MFE}([R, s_i, r_j, 0]) = +\infty$.
- **Case 3:** $S = \{s_i, t_k\} \cup S' \cup S''$ for some $t_k \neq r_j$, where S' (resp. S'') is the best structure for any $I' \prec [R', s_i, t_k, c]$ (resp. $I' \prec [R'', t_k, r_j, c]$), with R' being all strands between s and t in the polymer graph of S, and R'' being all strands between t and r.

Note that s and t are connected, thus in S' the connectivity bit will be set to 2. On the other hand, the connectedness of t and r (for structure S'') is by transitivity of connectivity determined by the connectedness between s and r, that is, c. We then have $\text{MFE}([R, s_i, r_j, c]) = E_{s_i,t_k} + \text{MFE}(]R', s_i, t_k, 2[) + \text{MFE}(]R'', t_k, r_j, c[)$.

□

Detailed conditions and edge cases. When we minimize over all subsets, the following conditions must be respected:

$$\begin{aligned} &\{s,t\} \subseteq R' \subseteq R \wedge 1 \leq k \leq |t| \wedge (k = |t| \rightarrow c \neq 1) \\ &\wedge (s = t \rightarrow (k > i + \theta \wedge R' = \{s\})) \\ &\wedge (r \in R' \rightarrow (t = r \wedge k < j \wedge R' = R \wedge c \neq 0)) \end{aligned}$$

We minimize over all possible triples (R', t, k). A set R' must clearly include s and t to form a valid interval and k must be a valid position of t. If s_i is paired to $t_{|t|}$, s and j are disconnected ($c \neq 1$). If $s = t$, we must respect θ and there is only one strand in R'. Finally, $r \in R'$ implies that s_i forms a base pair with some base of r (thus $t = r$ and $R' = R$), connectivity has to be allowed ($c \neq 0$) and t_k must be in the interval ($k < j$). These conditions are sufficient and match our algorithm.

When we minimize over two new inner strands (in the last case of $\bar{M}$), we clearly cannot choose the same strand for t and u, except if $|R| = 3$. Furthermore, we can clearly only minimize over new inner strands if such strands are still available. If $|R| \leq 3$, there may only be one available strand, or none at all, in which case the energy contribution is 0. We omit these edge cases in the presentation of the algorithm for the sake of readability.

4.5 Predicting *strand soup* interactions

We now consider the computational problem MFE Strand Soup Interaction. In comparison to above, we no longer need to keep track of the (exponentially many) subsets, or consider any strand association penalty since we require one single complex, but must enforce global connectivity of the strands set. Towards that goal, we introduce a *connectivity bit* c such as $c = 1$ indicate that the two outer strands have to be (transitively) connected in the corresponding interval, and $c = 0$ if they do not need to be connected (but can still be).

Let E_{s_i,r_j} be the energy contribution of pairing the i-th base of strand s to the j-th base of strand j, and let $M_s[i,j]$ be the classical single-stranded minimum free energy in the interval from i to j in strand s. Then the minimum free-energy (BP energy model) of a secondary structure over m strands subject to c, flanked by a suffix

$[i, |s|]$ of a strand s and a prefix $[1, j]$ of j, obeys:

$$M_{s_i,r_j,m,c} = \min \begin{cases} \overline{M}_{s_{i+1},r_j,m,c} \\ \min_k E_{s_i,s_k} + M_s[i+1,k-1] + \overline{M}_{s_{k+1},r_j,m,c} \\ \min_{\substack{t \in R \\ 1 \le k \le |t| \\ m'+m''=m-1}} E_{s_i,t_k} + \overline{M}_{s_{i+1},t_{k-1},m',0} + \overline{M}_{t_{k+1},r_j,m'',c} \\ \min_k E_{s_i,r_k} + \overline{M}_{s_{i+1},r_{k-1},m,0} + M_r[k+1,j] \end{cases} \tag{6}$$

with the following auxiliary table, responsible for the introduction of new strands whenever those identified by s and r have been entirely *consumed*:

$$\overline{M}_{s_i,r_j,m,c} = \begin{cases} \left.\begin{cases} \min_{t \in R} \overline{M}_{t_1,r_j,m-1,1} & \text{if } c = 0 \text{ and } m > 0 \\ M_r[1,j] & \text{if } c = 0 \text{ and } m = 0 \\ \infty & \text{if } c = 1 \end{cases}\right| & \text{if } i > |s| \\ \left.\begin{cases} \min_{t \in R} \overline{M}_{s_i,t_{|t|},m-1,1} & \text{if } c = 0 \text{ and } m > 0 \\ M_s[i,|s|] & \text{if } c = 0 \text{ and } m = 0 \\ \infty & \text{if } c = 1 \end{cases}\right| & \text{else if } j < 1 \\ M_{s_i,r_j,m,c} & \text{otherwise} \end{cases} \tag{7}$$

The minimum free energy can be finally computed by

$$E^*(R,m) = \min_{s,r \in R} M_{m-2,s_1,r_{|r|},1} \tag{8}$$

and the optimal secondary structure can be obtained through backtracking. We initialize $M_{1,s_i,s_j,2} = 0$ for all $j - i \le \theta$.

Theorem 7. *For any strand set R and number m of strands, the value found in $E^*(R,m)$, following its computation through Equations (6), (7) and (8), is the MFE of a connected secondary structure over m strands.*

Proof. We proceed by induction. We consider tuples of the form $(m, s, -i, r, j, c)$, and introduce an increasing lexicographic relation $\prec$ over tuples, defining a total order over such tuples. Notice that we negate the position of the left strand since we want to compute the rightmost positions first. We then hypothesize that, for any tuple $(m', s', -i', r', j', c')$ such that $(m', s', -i', r', j', c') \prec (m, s, -i, r, j, c)$, the following properties holds:

- $\overline{M}_{m',s'_{i'},r'_{j'},c'}$ contains the MFE of $m - \mathbb{1}_{i>|s|} - \mathbb{1}_{j<1}$ connected strands, starting and ending with s_i and r_j respectively, both of which may be empty. Additionally, if $c = 1$, the MFE is restricted to structures (transitively) connecting s_i to r_j;
- $M_{m',s'_{i'},r'_{j'},c'}$ contains the MFE of m connected strands, starting and ending with non-empty s_i and r_j respectively. Additionally, if $c = 1$, the MFE is restricted to structures (transitively) connecting s_i to r_j.

In both of the above cases, a $+\infty$ value is expected whenever constraints are overall unsatisfiable.

First, let us observe that base cases only concern $\overline{M}$, and let us discuss the correction of the equation in this context:

- If $i > |s|$ (s_i is actually empty), and $c = 1$ (s needs to be connected to r, but no such base pair has been formed at this stage), then $\overline{M}$ represents the MFE over an empty set of secondary structure, and should be set to ∞ as seen in the DP equation. The same reasoning holds when $j < 1$ (r_j empty), and $c = 1$;
- If $i > |s|$ (s_i empty), $m = 0$ (no strand left to insert) and $c = 0$ (no obligation to pair s_i to r_j), then the only energy of a structure stems from the MFE over r_j (if non-empty), found in $M_r[1, j]$ as correctly returned by the equation;
- If $j < 1$ (r_j empty), $m = 0$ (no strand left to insert) and $c = 0$ (no obligation to pair s_i to r_j), then the only energy of a structure stems from the MFE over s_i (if non-empty), found in $M_s[i, |s|]$ as correctly returned by the equation.

Next, we use the two induction hypotheses to show the correctness of the value found in $M_{m,s_i,r_j,c}$ and, following that, the correctness of $\overline{M}_{m,s_i,r_j,c}$. To show that $M_{m,s_i,r_j,c}$ is correct, let us observe that, within any secondary structure, the first position (i in strand s) is either unpaired, or paired to some position k within a strand:

- If i is left unpaired, the MFE is that of the (possibly empty) $[i+1, |s|]$ suffix of s. From the induction hypothesis, such an energy can be found in $\overline{M}_{m,s_{i+1},r_j,c}$, correctly computed since $(m, s, -(i+1), r, j, c) \prec (m, s, i, r, j, c)$;
- If i is paired to some k in the interval $[i + \theta + 1, |s|]$ of s, then the MFE of any such structure includes the contribution E_{s_i,s_k} of the base pair (i, k) in s, the MFE of the structure enclosed by the base pair ($\rightarrow M_s[i+1, k-1]$) and the MFE over m strands beginning with the remainder of s ($\rightarrow \overline{M}_{m,s_{k+1},r_j,c}$, correctly computed since $i < k + 1$);
- If i is paired to some k in a non-flanking strand t, then the MFE is obtained as the sum of the BP energy E_{s_i,t_k}, and the MFE contributions of two structures, being assigned m' and m'' strands such that $m' + m'' + 1 = m$, respectively enclosed ($\rightarrow \overline{M}_{m',s_{i+1},t_{k-1},0}$; $c = 0$ since a BP (i, k) already connects s and t) and preceded ($\rightarrow \overline{M}_{m'',t_{k+1},r_j,c}$; c is propagated since t may already be connected to r through s) by (s_i, t_k);
- If i is paired to some k in r, then the MFE consists in the energy E_{s_i,r_k} of the BP (s_i, r_k), augmented by the MFE over the remainder of r and s *under* (s_i, r_k) ($\rightarrow \overline{M}_{s_{i+1},r_{k-1},m,c}$) and the independent folding of the portion $[k+1, j]$ of r following r_k ($\rightarrow M_r[k+1, j]$).

These 5 cases can be verified to match the contributions in Equation (6). Moreover, the decomposition is complete, as it covers all possible outcomes for s_i. We conclude that minimizing over those yields the correct value, *i.e.* the $M_{m,s_i,r_j,c}$, and that the correctness of M and $\overline{M}$ for each $(m', s', -i', r', j', c') \prec (m, s, -i, r, j, c)$ implies the correctness of M for $(m, s, -i, r, j, c)$.

We are then only left to establish the correctness of $\overline{M}_{m,s_i,r_j,c}$, noting that its only difference in comparison with $M_{m,s_i,r_j,c}$ is its support for empty flanking regions. We focus on cases where one or several additional strands needs to be inserted and can be ($m > 0$):

- If s is fully depleted ($i > |s|$), then a strand t needs to be introduced to replace the leftmost flanking s. Since t is new it needs to be connected to r and, transitively, to each of the $m-1$ other strands that will be inserted by subsequent calls ($\to c = 1$). The MFE of such a structure can therefore be found in $\overline{M}_{t_1,r_j,m-1,1}$, correctly computed by the equation since $(m-1, t, -1, r, j, c) \prec (m, s, -i, r, j, c)$;
- Similarly, if r is fully depleted ($j < 1$), then a strand t needs to be introduced as the new rightmost flanking strand r. Since t is new it needs to be connected to s and the other $m-1$ other strands inserted by subsequent calls ($\to c = 1$). The MFE of such a structure is found in $\overline{M}_{s_i,t_{|t|},m-1,1}$, which correctly computed by the equation since $(m-1, s, -i, t, |t|, c) \prec (m, s, -i, r, j, c)$;
- If neither s nor r is depleted, then we are left to consider the structures over m connected strands, flanked by s_i and r_j, with connectivity bit c. The MFE of such a structure is found in $M_{m,s_i,r_j,c}$, correctly computed as established above.

Having already discussed base/terminal cases, we establish the completeness of the case decomposition and, in turn, on the correctness of the value found in $\overline{M}_{m,s_i,r_j,c}$. The induction step follows, and we finally conclude that the content of both $M_{m,s_i,r_j,c}$ and $\overline{M}_{m,s_i,r_j,c}$ match their specification.

The correctness of the MFE found in $E^*(R, m)$ follows from the choice of two globally-flanking strands s and r, left fully available (s_1 and $r_{|r|}$) while ensuring their connectivity ($c = 1$). For each (s, r) pair, the MFE of such structures can be found in $M_{m-2,s_1,r_{|r|},1}$, so the minimization computed in Equation (8), concluding our proof. □
